# PTPN22-CD45 dual phosphatase retrograde feedback enhances TCR signaling and autoimmunity

**DOI:** 10.1126/sciadv.adw2568

**Published:** 2025-09-05

**Authors:** Shen Yang, Eugenio Santelli, Carlos G. Gonzalez, Wade T. Johnson, Irene V. Choi, Chuling Zhuang, Myungja Ro, Leigh-Ana M. Rossitto, I-Shing Yu, Shu-Wha Lin, Yuan Zhan, Qinwei Chen, Jonathan D. Yoshihara, Daniel J. Wallace, Caroline A. Jefferies, Michifumi Yamashita, David J. Gonzalez, Richard I. Ainsworth, Nisarg J. Shah, Stephanie M. Stanford, Nunzio Bottini

**Affiliations:** ^1^ Department of Medicine, Altman Clinical and Translational Research Institute, University of California, San Diego, La Jolla, CA 92093, USA.; ^2^ Department of Medicine, Kao Autoimmunity Institute and Division of Rheumatology, Cedars-Sinai Medical Center, Los Angeles, CA 90048, USA.; ^3^ Department of Pharmacology, University of California, San Diego, La Jolla, CA 92093, USA.; ^4^ Skaggs School of Pharmacy and Pharmaceutical Sciences, University of California, San Diego, La Jolla, CA 92093, USA.; ^5^ Department of Chemical and Nano Engineering, University of California, San Diego, La Jolla, CA 92093, USA.; ^6^ Laboratory Animal Center, College of Medicine, National Taiwan University, Taipei 10048, Taiwan.; ^7^ Department of Clinical Laboratory Sciences and Medical Biotechnology, College of Medicine, National Taiwan University, Taipei 10048 Taiwan.; ^8^ Department of Laboratory Medicine, National Taiwan University Hospital, College of Medicine, National Taiwan University, Taipei 10048, Taiwan.; ^9^ Department of Pathology and Laboratory Medicine, Cedars-Sinai Medical Center, Los Angeles, CA 90048, USA.

## Abstract

Protein tyrosine phosphatase nonreceptor type 22 (PTPN22) is encoded by a gene strongly associated with lupus and other autoimmune diseases. PTPN22 regulates T cell receptor (TCR) signaling through dephosphorylation of the kinases lymphocyte-specific protein tyrosine kinase (LCK) and zeta-chain–associated protein kinase 70 (ZAP70). The regulation of PTPN22 remains poorly understood. Here, we identify PTPN22 Ser^449^ as a protein kinase A phosphorylation site, which is triggered by TCR engagement and is hyperphosphorylated in lupus peripheral blood cells. PTPN22 Ser^449^ phosphorylation selectively lowered the affinity of PTPN22 for ZAP70 versus LCK but also indirectly suppressed inhibitory LCK Tyr^192^ phosphorylation through a ZAP70-CD45 signaling axis. The resulting dephosphorylation of LCK Tyr^192^ not only enhanced TCR signaling but also modulated pathway activation downstream the TCR. In vivo loss of PTPN22 Ser^449^ phosphorylation reduced T cell responses and suppressed experimental lupus nephritis. These results suggest that PTPN22 Ser^449^ phosphorylation promotes a CD45-mediated retrograde ZAP70-LCK feedback loop that enhances T cell responses and promotes autoimmunity.

## Teaser

PTPN22 Ser^449^ phosphorylation enables ZAP70-CD45-LCK feedback loop that boosts T cell activation and contributes to autoimmunity.

## INTRODUCTION

Dysregulated T cell receptor (TCR) signaling is a critical contributor to systemic lupus erythematosus (SLE) pathophysiology and contributes to the breakdown of immune tolerance and the amplification of pathogenic immune responses (*1*). Studies from multiple laboratories have established that SLE T cells display abnormal TCR signaling, which affects downstream T cell differentiation and contributes to the immunopathology of disease (*2*, *3*). Abnormalities in the composition of the TCR signaling complex, calcium signaling, and endosomal trafficking have been described in SLE T cells (*3*–*5*).

Engagement of TCR triggers a highly regulated cascade of phosphorylation events via the concerted action of protein kinases and phosphatases (*6*). Zeta chain–associated protein kinase 70 (ZAP70) and the Src family kinase lymphocyte-specific protein tyrosine kinase (LCK) are protein tyrosine kinases whose catalytic activities are central to early T cell signaling (*7*, *8*). Upon TCR engagement, with or without coreceptor involvement, activated LCK phosphorylates TCR CD3 and zeta chains leading to ZAP70 recruitment and its LCK-mediated phosphorylation on several residues, including Tyrosine 319 (Tyr^319^) (*9*). Binding of the LCK Src homology 2 (SH2) domain to Tyr^319^-phosphorylated ZAP70 then sustains the activities of both kinases in a positive feedback mechanism that promotes downstream signaling events (*10*). LCK catalytic activity is controlled by its SH2 and SH3 and by phosphorylation at Tyr residues (*11*). Phosphorylation of LCK Tyr^394^ in the so-called activation loop and Tyr^505^ near the C terminus stimulate and suppress LCK activity, respectively. Tyr^394^ is trans-autophosphorylated by LCK and is dephosphorylated by the SH2 domain–containing phosphatase-1 (SHP-1) and protein tyrosine phosphatase nonreceptor type 22 (PTPN22) (*12*, *13*). Tyr^505^ is phosphorylated by C-terminal Src kinase (CSK) and dephosphorylated by CD45 (*14*). In addition, a recent study revealed a role for an additional phosphorylation site of LCK on Tyr^192^, which is thought to be regulated by interleukin-2 (IL-2) inducible Tec kinase (ITK), enabling a negative feedback loop involving reduced access to the inhibitory phospho-Tyr^505^ (pTyr^505^) by CD45 after TCR activation (*14*, *15*). It currently remains unknown which PTP dephosphorylates LCK-pTyr^192^.

PTPN22 is a hematopoietic cell–specific cytosolic protein tyrosine phosphatase acting as a key negative regulator of early TCR signaling through dephosphorylation of LCK and ZAP70. PTPN22 potentially has additional, less well-characterized substrates in T cells (*16*). In other immune cells, it regulates signaling pathways downstream of the type 1 interferon (IFN) and other receptors (*17*, *18*). PTPN22 consists of three domains: an N-terminal, well-characterized classic PTP catalytic domain (amino acids 1 to 300); an interdomain (301 to 600), which may have regulatory functions but whose mechanism of action remains poorly understood; and a C-terminal domain (601 to 807), which carries four proline-rich motifs (P1 to P4) and facilitates protein-protein interactions (*19*, *20*). A R620W polymorphism (C1858T) in the P1 motif of PTPN22 has emerged as a major autoimmunity risk factor, and a strong association between PTPN22 R620W and SLE has been reported by many studies (*21*). The functional effect of the W620 mutation in various T cells remains to be fully clarified; however, studies in knock-in (KI) mice generally pointed to the autoimmune-associated mutant causing loss or change in function of the molecule (*16*).

Despite the critical role of PTPN22 in T cell biology and autoimmune diseases, its regulatory mechanisms during TCR signaling and disease progression remain inadequately understood. We and others have identified one regulatory tyrosine (Tyr^536^) and two serine (Ser^35^ and Ser^751^) PTPN22 phosphorylation sites (*22*–*24*); however, phosphorylation of PTPN22 has not been assessed in autoimmunity yet. Here, we report that Ser^449^ within the PTPN22 interdomain is an unrecognized protein kinase A (PKA) target site–induced post–TCR stimulation, and its phosphorylation is pronouncedly elevated in peripheral blood mononuclear cells (PBMCs) from patients with SLE. The biochemical characterization of the effects of phospho-Ser^449^ on early TCR signaling, coupled with the assessment of KI mice, uncovered previously unreported PTPN22-controlled retrograde feedback loop, which is activated by ZAP70 and impairs dephosphorylation of LCK Tyr^192^ by CD45, thus promoting TCR signaling and autoimmunity.

## RESULTS

### Ser^449^ in the interdomain of PTPN22 is an unidentified PKA target site and is hyperphosphorylated in SLE PBMCs

**Fig. 1.**
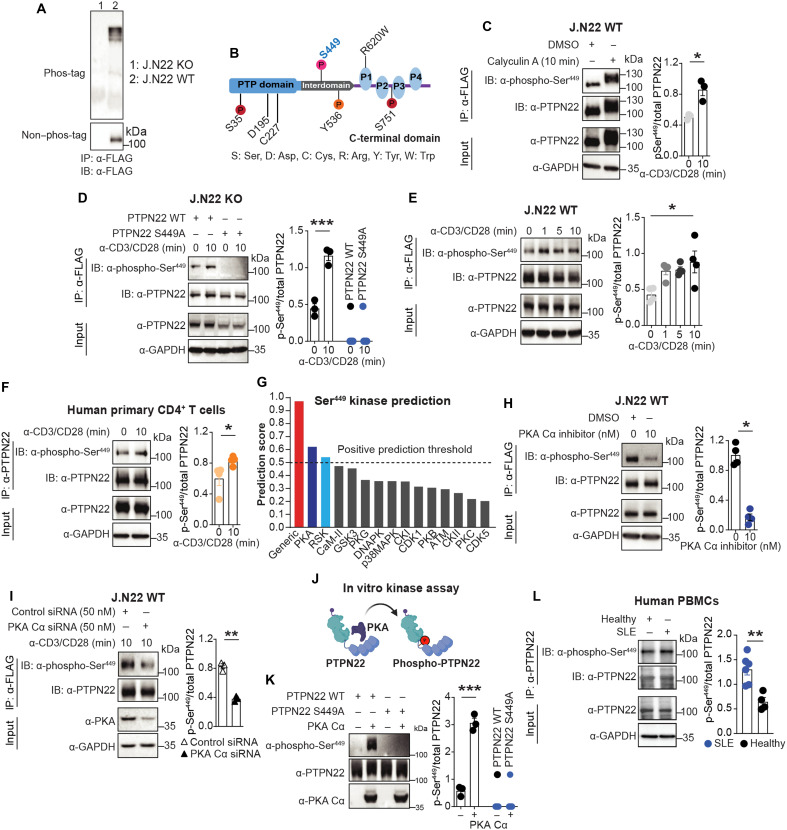
Ser^449^ of PTPN22 is targeted by PKA and hyperphosphorylated in SLE PBMCs. (**A**) Analysis of PTPN22 phosphorylation by Phos-tag SDS-PAGE and Western blotting in J.N22 KO and J.N22 WT Jurkat cells. Non–phos-tag gel served as control. (**B**) Schematic view of the PTPN22 structure with key catalytic residues, known phosphorylation sites including the Ser^449^ site in the interdomain and the R620W variation. (**C** to **F**) Ser^449^ phosphorylation was assessed by Western blotting following immunoprecipitation of PTPN22 from J.N22 WT Jurkat cells treated with calyculin A (C), or in J.N22 KO cells expressing FLAG-tagged WT or S449A PTPN22, with or without 10-min TCR stimulation (D), or in J.N22 WT cells after 0-, 1-, 5-, and 10-min TCR stimulation (E), or in primary human CD4^+^ effector T cells with or without 10-min TCR stimulation (F). (**G**) NetPhos hits with prediction score > 0.5, PKA (0.621) and RSK (0.541) were predicted Ser^449^ kinases. “Generic” indicates overall prediction of Ser^449^ as a phosphorylation site. (**H** and **I**) Effect of PKA Cα inhibitor [(H) H89 2HCl] or PKA Cα siRNA (I) on Ser^449^ phosphorylation in J.N22 WT cells after TCR stimulation. (**J** and **K**) In vitro PKA kinase assay using purified recombinant WT or S449A PTPN22 from HEK293T cells as substrates. (**L**) Ser^449^ phosphorylation in primary patients with SLE (*n* = 6) versus healthy controls (*n* = 4) PBMCs was analyzed by Western blotting following immunoprecipitation of PTPN22. Representative blots are shown (left). Histograms show comparison of Ser^449^ phosphorylation levels between stimulated/treated and control groups (right). Data are presented as means ± SEM and are from three (A, C, D, I, and K) or four (E, F, and H) independent experiments. Statistical significance was assessed by two-tailed Mann-Whitney test (C, F, H, I, and L), two-way analysis of variance (ANOVA) followed by Bonferroni’s post hoc test (D and K), or one-way ANOVA followed by Dunn’s post hoc test (E). *P < 0.05, **P < 0.01, ***P < 0.001. (J) Created with BioRender (*69*).

PTPN22 phosphorylation status in immune cells has only been partially characterized (*24*). To further explore PTPN22 phosphorylation in T cells, we took advantage of a Jurkat CD4^+^ T cell line carrying PTPN22 with a KI 3× FLAG tag [J.N22 wild-type (WT)] (*24*). PTPN22 was immunoprecipitated from these cells after TCR engagement and analyzed by Phos-tag SDS–polyacrylamide gel electrophoresis (SDS-PAGE) followed by Western blotting with an anti-FLAG antibody (Fig. 1A). This analysis revealed a range of migration rates of PTPN22 molecules, likely due to variable phosphorylation of multiple sites in the Jurkat T cells PTPN22 pool. Next, the phosphorylation sites of PTPN22 purified from TCR-stimulated Jurkat T cells (fig. S1, A and B) were determined using mass spectrometry (MS). Multiple peptides containing phosphorylation at residue Ser^449^ (TKS + 79.66331TPFELIQQR) were detected in stimulated Jurkat T cells (fig. S1C). This site is located within the PTPN22 interdomain (Fig. 1B) and is conserved among various species (fig. S1D). We generated a phospho-specific antibody recognizing PTPN22 phosphorylated on Ser^449^ whose reactivity to PTPN22 immunoprecipitated from WT cells was enhanced more than twofold by cell treatment with the serine/threonine phosphatase inhibitor calyculin A (Fig. 1C) or stimulation through the TCR (Fig. 1D). The anti–phospho-Ser^449^ antibody showed no reactivity to PTPN22 mutated at Ser^449^ to Ala (Fig. 1D). In a time course (1 to 10 min) of TCR engagement, PTPN22 Ser^449^ phosphorylation was induced at all time points (Fig. 1E). Moreover, PTPN22 Ser^449^ phosphorylation is present in unstimulated T cells and could be further induced in primary human T cells (Fig. 1F). These data suggest that Ser^449^ of PTPN22 undergoes TCR-increased phosphorylation in human T cells.

To address the kinase(s) mediating PTPN22 Ser^449^ phosphorylation, we first examined protein kinase C (PKC) as a candidate since PTPN22 undergoes PKC-mediated phosphorylation at Ser^35^ and Ser^751^ (*23*, *24*). We used an array of PKC inhibitors; however, none influenced TCR-induced PTPN22 Ser^449^ phosphorylation (fig. S1E). Next, we used the NetPhos kinase prediction software to analyze the amino acid sequence surrounding PTPN22 Ser^449^ and found PKA and ribosomal S6 kinase (RSK) as the top two candidate PTPN22 Ser^449^ kinases (Fig. 1G). However, while an inhibitor of PKA catalytic subunits (PKA Cs) blocked TCR-induced phosphorylation of PTPN22 Ser^449^ (Fig. 1H), an RSK inhibitor had no effect (fig. S1F). PKA C-alpha (PKA Cα) is the major cytosolic PKA C in Jurkat and primary T cells (*25*, *26*). PTPN22 Ser^449^ phosphorylation was dramatically reduced when *PRKACA* was knocked down by small interfering RNA (siRNA) (Fig. 1I). PKA Cα was also coprecipitated with PTPN22 from Jurkat cells, and the interaction did not require the Ser^449^ residue (fig. S1G). When we examined PKA activity in T cells by evaluating the phosphorylation of its key downstream mediator cyclic adenosine monophosphate–responsive element–binding protein (CREB) (*27*), we found that it was markedly promoted by TCR stimulation (fig. S1H), with a time course compatible with the one observed for PTPN22-Ser^449^. Last, to verify that PKA Cα can directly phosphorylate PTPN22 at Ser^449^, purified FLAG-tagged PTPN22 WT and S449A proteins were incubated with PKA Cα in vitro (Fig. 1J). Incubation of PTPN22 WT—but not the S449A mutant—with PKA resulted in strong signal detected by Western blotting with the phospho-Ser^449^ antibody (Fig. 1K). Because *PTPN22* is a major SLE genetic risk factor (*21*) and disease-activity dependent increase in free cytosolic PKA Cs has been observed in lupus T cells (*28*), we decided to evaluate the phosphorylation of PTPN22 Ser^449^ in PBMCs from patients with SLE with active disease (SLE disease activity index, SLEDAI >2) (*29*) and healthy controls (see patient and control data in table S1). Figure 1L shows that the phosphorylation levels of Ser^449^ were noticeably up-regulated in PBMCs from patients with SLE. Together, our data indicate that Ser^449^ is a TCR-inducible PKA phosphorylation site up-regulated in SLE immune cells.

### Ser^449^ phosphorylation inhibits PTPN22 functions and enhances TCR signaling

**Fig. 2.**
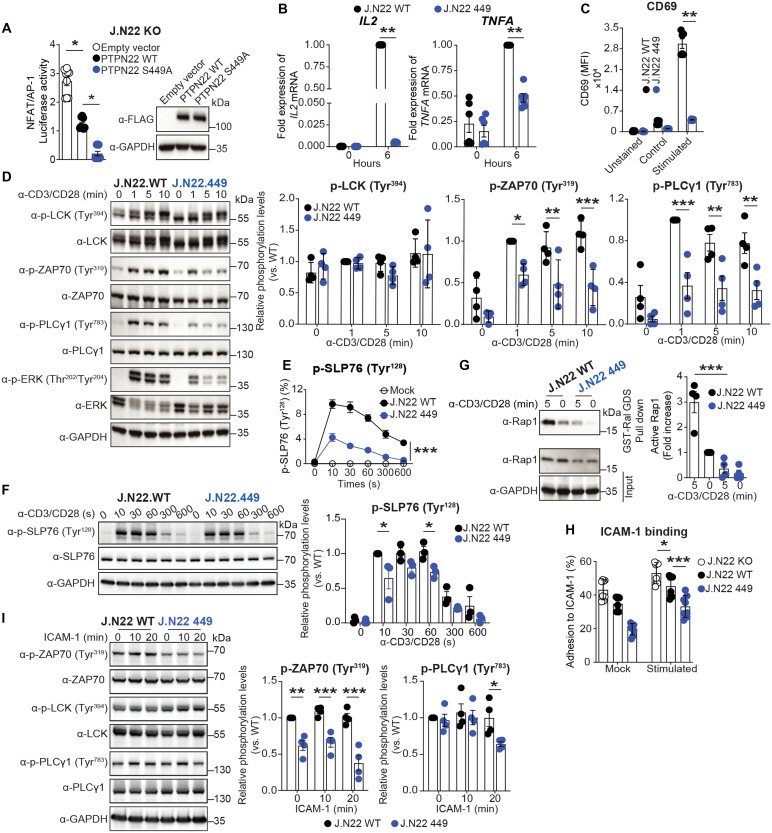
Ser^449^ phosphorylation inhibits PTPN22 functions during TCR signaling. (**A**) Evaluation of TCR signaling–dependent PTPN22 activity by dual-luciferase reporter assay in J.N22 KO cells. Luciferase activity was measured (left, with pooled replicates) and normalized to the amount of PTPN22 relative to that of glyceraldehyde-3-phosphate dehydrogenase (GAPDH) as assessed by Western blotting (right). (**B**) Real-time RT-PCR analysis of *IL2* and *TNFA* mRNA expression in J.N22 WT and J.N22 449 cells. (**C**) TCR-induced CD69 activation in J.N22 WT and J.N22 449 cells analyzed by flow cytometry and evaluated by median fluorescence intensity (MFI) of cells. (**D**) Protein phosphorylation induced by TCR stimulation in J.N22 WT and J.N22 449 cells analyzed by Western blotting. (**E** and **F**) TCR stimulation–induced SLP-76 Tyr^128^ phosphorylation in J.N22 WT and J.N22 449 cells assessed by flow cytometry (E) or Western blotting (F) at time points between 0 and 10 min. (**G**) Endogenous Rap1 activity in J.N22 WT and J. N22 449 cells assessed through RAS-like guanine nucleotide dissociation stimulator (Ral GDS) pull down was detected by immunoblotting and normalized to WT at 0 min. (**H**) The percentage of attached J.N22 WT or J.N22 449 cells to immobilized ICAM-1 was assessed following stimulation with or without antibodies against human CD3/CD28 and with plate-coated ICAM-1. (**I**) Integrin-mediated outside-in signaling in J.N22 WT and J.N22 449 cells was assessed by measuring protein phosphorylation as shown after stimulation with ICAM-1 for 0, 10, and 20 min. Representative blots are shown. Histogram shows quantification of phosphorylated protein normalized to total protein (D, F, and I). Data are presented as means ± SEM and are from three (A, E, and F), six (B and C), four (D, G, and I), or eight (H) independent experiments. Statistical significance was assessed by one-way ANOVA followed by Dunn’s post hoc test (A), Kolmogorov-Smirnov test (B), and two-way ANOVA followed by Bonferroni’s post hoc test (C to I). *P < 0.05, **P < 0.01, ***P < 0.001.

To understand the potential effects of Ser^449^ phosphorylation on the function of PTPN22 in T cells, we leveraged a nuclear factor of activated T cells and activator protein-1 (NFAT/AP-1) luciferase reporter activity, which is commonly used to quantitate signaling downstream TCR activation (*30*). Expression of S449A PTPN22 led to notably reduced TCR-induced luciferase reporter activation compared to similar levels of WT PTPN22 (Fig. 2A) suggesting that S449A PTPN22 behaves as a gain-of-function mutant. The R620W genetic variation in the P1 motif of PTPN22 impairs the interaction of PTPN22 with CSK, tumor necrosis factor receptor-associated factor 3 (TRAF3), and other TCR effectors; however, we found no substantial impairment in Ser^449^ phosphorylation between R620 and W620 PTPN22 upon TCR stimulation (fig. S2A). Further suppression of NFAT/AP-1 luciferase activity was also observed in cells expressing the double W620 S449A mutant compared to W620, indicating that Ser^449^ phosphorylation inhibits the function of both R620 and W620 PTPN22 variants (fig. S2B). Moreover, diminished expression of CSK had no impact on Ser^449^ phosphorylation of PTPN22 (fig. S2C), overall suggesting that the P1 domain of PTPN22 is not involved in the regulation of Ser^449^ phosphorylation.

To avoid the confounding effects of endogenous WT PTPN22, we generated a Jurkat PTPN22 S449A KI line by CRISPR-Cas9–mediated genome editing (J.N22 449; fig. S2D). The phosphorylation-null mutation was confirmed by various methods (table S2 and fig. S2, E to H). TCR-induced IL-2 and tumor necrosis factor–α (TNF-α) mRNA expression and activation of CD69 were decreased in J.N22 449 cells (Fig. 2, B and C, and fig. S2I), suggesting attenuated downstream T cell activation. We next assessed early phosphorylation events after TCR engagement and found markedly diminished TCR-triggered tyrosine phosphorylation of ZAP70 Tyr^319^, of ZAP70 substrate SH2 domain–containing leukocyte protein of 76 kDa (SLP-76) Tyr^128^ and ZAP70 downstream adaptor phospholipase C ϒ (PLCϒ) Tyr^783^ in J.N22 449 cells compared to WT cells. However, phosphorylation of another well-established PTPN22 substrate, LCK Tyr^394^, remained unaffected (Fig. 2, D to F, and fig. S2J).

PTPN22 has been implicated with integrin lymphocyte function–associated antigen 1 (LFA-1) (*31*). TCR triggers rapid integrin-mediated adhesion of T cells involving “inside-out” signals and “outside-in” signaling mediated by ZAP70 (*32*, *33*). TCR-induced activation of RAS-associated protein 1 (Rap1) was strongly inhibited (Fig. 2G), and decreased cell adhesion to the LFA-1 ligand intercellular adhesion molecule 1 (ICAM-1) was observed in J.N22 449 cells compared to WT or knock-out (KO) cells (Fig. 2H). Furthermore, J.N22 449 cells showed reduced phosphorylation of ZAP70 upon ICAM-1 binding, pointing to impaired integrin outside-in signaling in these cells (Fig. 2I). Together, these results indicate that Ser^449^ phosphorylation reduces the ability of PTPN22 to dephosphorylate ZAP70, which promotes TCR signaling downstream ZAP70.

### Ser^449^ phosphorylation impairs PTPN22 binding to ZAP70

We next sought to determine whether Ser^449^ phosphorylation inhibits PTPN22’s phosphatase activity and found that the activity of PTPN22 immunoprecipitated from TCR-stimulated S449A cells was unchanged compared to WT when using 6,8-difluoro-4-methylumbelliferyl phosphate (DiFMUP) as a substrate (fig. S3A). Previously, we reported that PTPN22 Ser^751^ phosphorylation in the C-terminal domain extends PTPN22 half-life in T cells (*24*), prompting us to determine whether Ser^449^ phosphorylation also regulates PTPN22’s half-life. However, the abundance of S449A PTPN22 remained akin to WT in time courses after cycloheximide treatment (fig. S3B).

**Fig. 3.**
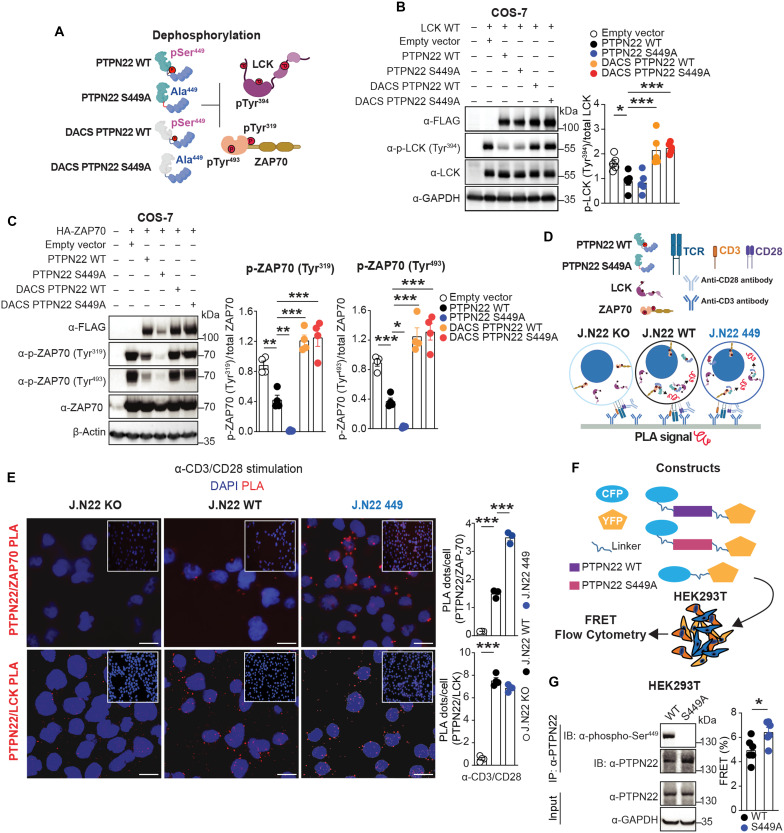
Ser^449^ phosphorylation impairs PTPN22 binding to ZAP70. (**A**) Schematic illustration of PTPN22 variants and dephosphorylation of substrates LCK and ZAP70. (**B**) Immunoblotting analysis of COS-7 cells cotransfected with untagged LCK WT and 3× FLAG tagged WT, S449A, DACS WT, or DACS S449A PTPN22. Histogram shows quantification of the phospho-Tyr^394^ LCK /total LCK ratio (right). (**C**) Dephosphorylation of ZAP70 in COS-7 cells cotransfected with hemagglutinin (HA)–tagged ZAP70 and 3× FLAG tagged WT, S449A, DACS WT, or DACS S449A PTPN22 assessed by immunoblotting. Histograms show quantification of the phospho-Tyr^319^ or Tyr^493^ ZAP70 /total ZAP70 ratio. (**D**) PLA in J.N22 KO, WT and 449 cells stimulated by plate-bound anti-CD3 and soluble anti-CD28 antibodies for 30 min. (**E**) PLA was performed between PTPN22 and ZAP70 or LCK using mouse anti-FLAG and rabbit anti-ZAP70 or -LCK antibodies. Images of PLA (red, left) and quantification of PLA are shown (right). Scale bars, 10 μm. (**F**) Schematic model of PTPN22 intracellular FRET. (**G**) FRET measurements of PTPN22 conformation dynamics by flow cytometry. PTPN22 Ser^449^ phosphorylation and expression of PTPN22 FRET fusion proteins was evaluated by immunoprecipitation and Western blot analysis (left). FRET was assessed by adjusting the gates to identify cells that are negative in transfected with CFP-PTPN22 only, PTPN22-YFP only, CFP-PTPN22, and PTPN22-YFP. Histogram shows quantification of FRET % (right). Data are presented as means ± SEM and from three to five (B, C, and E) or six (G) independent experiments. Representative blots are shown (left). Statistical significance was assessed by one-way ANOVA followed by Dunnett’s post hoc test (B, C, and E), or two-tailed Mann-Whitney test (G). *P < 0.05, **P < 0.01, ***P < 0.001. (A) and (D) created with BioRender (*69*).

As TCR-induced phosphorylation of ZAP70 but not LCK was notably reduced in J.N22 449 cells, we next explored whether PTPN22-pSer^449^ differentially regulates the ability of PTPN22 to dephosphorylate its two main early TCR signaling substrates. After confirming that PTPN22 Ser^449^ is highly phosphorylated in COS-7 cells (fig. S3C), we assessed dephosphorylation of LCK in COS-7 cells coexpressing LCK with WT or S449A PTPN22 or with the catalytically inactive D195A/C227S (DACS) PTPN22 mutants (Fig. 3A). Phosphorylation of LCK Tyr^394^ decreased similarly in cells expressing WT or S449A PTPN22 compared to vector alone, while the DACS WT or DACS S449A PTPN22 mutants had no effect (Fig. 3B). Similar results were obtained using human embryonic kidney (HEK) 293T cells (fig. S3D). Figure 3C shows that phosphorylation of ZAP70 Tyr^319^ and Tyr^493^ was dramatically reduced in COS-7 cells coexpressing S449A PTPN22 compared with WT, and this difference was abolished in cells expressing DACS mutants of WT or S449A PTPN22. Consistent with the high stoichiometry of Ser^449^ phosphorylation observed in COS-7 cells, phospho-tyrosine mimetic PTPN22 S449E or S449D variants did not cause further decreases of ZAP70 phosphorylation in this system compared to WT PTPN22 (fig. S3E).

Distinct effects of PTPN22 Ser^449^ phosphorylation on LCK versus ZAP70 suggested that Ser^449^ phosphorylation differentially modulates PTPN22-binding affinity to these two substrates. Thus, PTPN22-LCK and -ZAP70 proximity ligation assays (PLAs) in J.N22 KO, J.N22 WT, and J.N22 449 Jurkat cells were performed, and revealed a substantial increase in the molecular colocalization of S449A PTPN22 with ZAP70 after T cell activation, but not with LCK (Fig. 3, D and E). To investigate whether the interaction between PTPN22 and ZAP70 is similarly regulated by S449A PTPN22 downstream ICAM-1 stimulation, PLA assay was also performed. The results demonstrated an increased molecular colocalization between PTPN22 and ZAP70 in J.N22 449 Jurkat cells (fig. S3, F and G). Considering the location of Ser^449^, which is situated in the more loosely ordered interdomain region of PTPN22 (*16*), one potential explanation for the above observation is that Ser^449^ phosphorylation contributes to a conformational change in PTPN22 after TCR engagement. To explore this, we used intramolecular Förster resonance energy transfer (FRET) detected by flow cytometry to monitor the dynamic conformation of PTPN22 in HEK293T transfected cells (Fig. 3, F and G). We observed enhanced FRET signal in cells expressing S449A PTPN22 compared with WT (Fig. 3G), suggesting that Ser^449^ phosphorylation affects the intracellular conformation of PTPN22. We conclude that PTPN22 phospho-Ser^449^ selectively promotes TCR-induced proximity to and dephosphorylation of ZAP70 versus LCK (and perhaps other PTPN22 substrates in T cells), likely through an effect on the interdomain-regulated PTPN22 conformation.

### Mutation of PTPN22 Ser^449^ elevates LCK Tyr^192^ phosphorylation

**Fig. 4.**
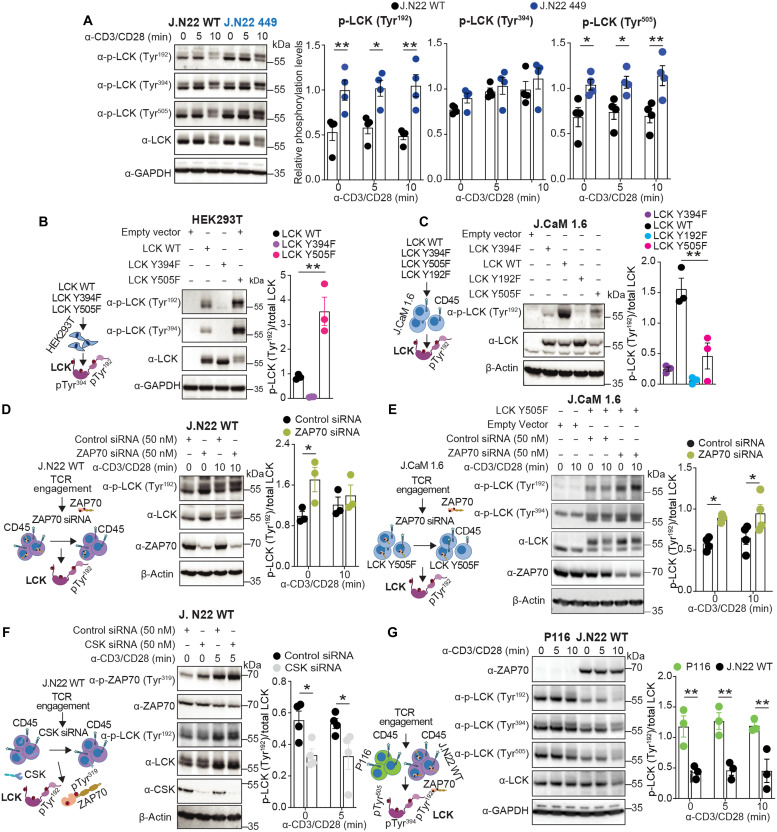
Mutation of PTPN22 Ser^449^ elevates LCK Tyr^192^ phosphorylation. (**A**) Immunoblotting analysis of phospho-proteins in total lysates of TCR stimulated J.N22 WT and J.N22 449 cells. Blots show LCK phosphorylation (Tyr^192^, Tyr^394^, or Tyr^505^) induced by TCR stimulation. (**B** and **C**) LCK phosphorylation in HEK293T (B) or J.CaM1.6 (C) cells transfected with LCK variants. (**D**) TCR-induced LCK Tyr^192^ phosphorylation in ZAP70 knockdown WT cells. (**E**) LCK phosphorylation in J.CaM1.6 cells transfected with LCK Y505F variant and subjected to ZAP70 knockdown after 10-min TCR stimulation. (**F**) LCK Tyr^192^ and ZAP70 Tyr^319^ phosphorylation in CSK knockdown WT cells. (**G**) Immunoblotting analysis of LCK phosphorylation in P116 cells induced by TCR stimulation. Data are presented as means ± SEM and are from four (A, E, and F) or three (B to D, and G) independent experiments. Representative blots are shown (left), and histograms show quantification of phosphorylated LCK normalized to total protein (right). Statistical significance was assessed by two-way ANOVA followed by Bonferroni’s post hoc test (A and D to G), and one-way ANOVA followed by Dunnett’s post hoc test (B and C). *P < 0.05, **P < 0.01. Schematics in (B) to (G) created with BioRender (*69*).

Intrigued by the selective effect of the PTPN22 S449A mutation on ZAP70 versus LCK-activating phosphorylation sites, we next turned our attention to other LCK phosphorylation sites in J.N22 449 cells. We thus assessed the three major LCK Tyr phosphorylation sites in an independent set of experiments. While we confirmed that phospho-Tyr^394^ was unaffected, we found that LCK Tyr^192^ and Tyr^505^ phosphorylation were up-regulated in resting as well as stimulated J.N22 449 cells (Fig. 4A). A potent and selective ITK inhibitor (*34*) did not block LCK Tyr^192^ phosphorylation (fig. S4A), overall ruling out ITK as a major LCK-Tyr^192^ kinase in our cells. Since ZAP70 had also been previously excluded as an LCK-Tyr^192^ kinase (*35*), we next hypothesized that Tyr^192^ could be an autophosphorylation site of LCK. We expressed LCK mutated at its various Tyr phosphorylation sites in HEK293T cells—which lack ITK—and in J.CaM1.6 cells, a derivative of Jurkat that lacks LCK (*36*) but expresses ITK. LCK Tyr^192^ phosphorylation was nearly absent in HEK293T cells expressing the inactive LCK Y394F mutant but was substantially enhanced in cells expressing the hyperactive LCK Y505F mutant (Fig. 4B), suggesting that LCK Tyr^192^ can be phosphorylated by active LCK. In J.CaM1.6 cells, LCK Tyr^192^ phosphorylation was again substantially reduced by the LCK Y394F mutation; however, the LCK Y505F mutant also displayed decreased Tyr^192^ phosphorylation when compared to WT LCK (Fig. 4C). Comparing these results, we suspected that LCK Tyr^192^ phosphorylation is under control of a T cell–expressed kinase or phosphatase that is absent in HEK293T cells and respectively inhibited or induced by TCR signaling. We decided to initially test this hypothesis by blocking signaling downstream ZAP70 and observed that Tyr^192^ phosphorylation was elevated in J.N22 WT cells subjected to knockdown of ZAP70 (Fig. 4D). Knockdown of ZAP70 also substantially increased LCK Tyr^192^ phosphorylation in JCaM 1.6 cells expressing LCK Y505F (Fig. 4E), while knockdown of CSK—which enhances TCR signaling—decreased LCK Tyr^192^ phosphorylation in J.N22 WT cells (Fig. 4F). These data suggest that TCR-induced ZAP-70 activity inhibits LCK Tyr^192^ phosphorylation through a retrograde feedback mechanism. We next assessed LCK phospho-Tyr^192^ in P116 T cells, a derivative of Jurkat lacking ZAP70 (*37*), and observed that it was highly enhanced in resting or TCR-stimulated conditions. Notably, phosphorylation of LCK Tyr^394^ and Tyr^505^ were also increased in P116 cells (Fig. 4G) (*37*). These data rule out ZAP70 and ITK as major LCK Tyr^192^ kinases and suggest that LCK activity and a T cell–expressed phosphatase induced by TCR signaling are necessary, respectively, for LCK Tyr^192^ phosphorylation and its down-regulation induced by low or absent ZAP70 activity.

### ZAP70-CD45 axis regulates LCK Tyr^192^ phosphorylation

**Fig. 5.**
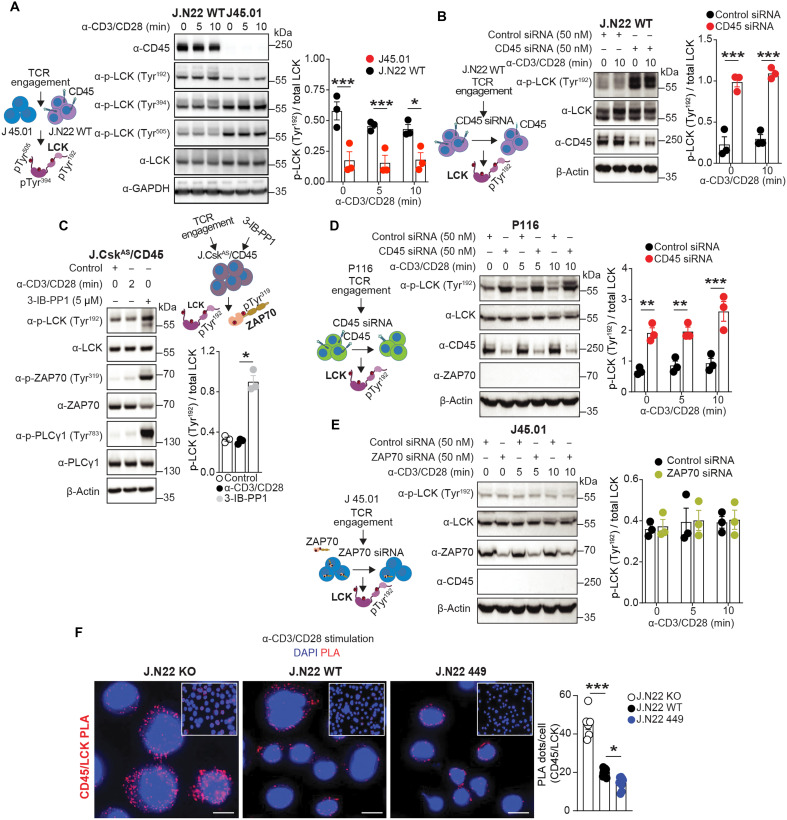
ZAP70-CD45 axis regulates LCK Tyr^192^ phosphorylation. (**A**) TCR stimulation–induced LCK phosphorylation in J45.01 cells. (**B**) TCR stimulation–induced LCK phosphorylation in CD45 knockdown WT cells. (**C**) Phosphorylation of LCK Tyr^192^ after CSK inhibition in CD45 deficient cells. J.Csk^AS^/CD45 cells were treated with antibodies against CD3/CD28 or the Csk^AS^ inhibitor (5 μM 3-IB-PP1) for 2 min (left). (**D** and **E**) LCK Tyr^192^ phosphorylation in CD45 knockdown P116 cells (D) or in ZAP70 knockdown J45.01 cells (E) after TCR stimulation for 0, 5, and 10 min. Representative blots are shown (left). Histograms show quantification of phospho-Tyr^192^ LCK to total protein ratio (right). (**F**) PLA assay in J.N22 KO, WT, and 449 cells stimulated with antibodies against human CD3/CD28 for 30 min. PLA was performed between CD45 and LCK using mouse anti-LCK and rabbit anti-CD45 antibodies. Representative images of PLA (red, left) and quantification of PLA are shown (right). Scale bars, 10 μm. Data are presented as means ± SEM and are from three (A to F) independent experiments. Statistical significance was assessed by two-way ANOVA followed by Bonferroni’s post hoc test (A, B, D, and E) and by one-way ANOVA followed by Dunnett’s post hoc test (C and F). *P < 0.05, **P < 0.01, ***P < 0.001. Schematics in (A) to (E) created with BioRender (*69*).

Initially, we hypothesized that PTPN22 might be the LCK Tyr^192^ phosphatase. However, deletion of PTPN22 enhances Tyr^394^ phosphorylation (*17*), and we found reduced Tyr^192^ phosphorylation in J.N22 KO T cells (fig. S4B). Overexpression of SH2-containing SHP-1 and SHP-2, which both have roles in TCR signaling (*38*) did not affect LCK Tyr^192^ phosphorylation in WT cells (fig. S4C). We next hypothesized that CD45 could serve as an LCK Tyr^192^ phosphatase and assessed LCK Tyr^192^ phosphorylation in J45.01 cells, a Jurkat derivative lacking CD45 (*39*). As CD45 is also known to dephosphorylate LCK Tyr^505^ and Tyr^394^, a regulation of CD45 function by ZAP70 could explain the triple-site hyperphosphorylation of LCK observed in P116 cells (Fig. 4G). However, Tyr^192^ phosphorylation was drastically reduced in J45.01 cells compared to J.N22 WT cells upon TCR stimulation (Fig. 5A). To rule out that the profound obliteration of LCK activity caused by total lack of CD45 in J45.01 had prevented the autophosphorylation of Tyr^192^, we next assessed LCK Tyr^192^ phosphorylation in WT cells in which CD45 was only partially knocked down by siRNA. In a notable reversal of the result obtained in J45.01 cells, LCK Tyr^192^ phosphorylation was markedly increased in WT cells when CD45 was knocked down (Fig. 5B). We confirmed that CD45 expression was equal in J.N22 WT and J.N22 449 cells (fig. S4D). As CD45 knockdown tends to lower LCK activity, these data suggest a potential dominant role for CD45 in control of LCK Tyr^192^ phosphorylation. Consistent with this hypothesis, when we expressed LCK Y505F in JCaM 1.6 cells subjected to CD45 knockdown, we observed a strong induction of LCK Tyr^192^ phosphorylation (fig. S4E). As phosphorylation at Tyr^505^ can be reduced by inhibiting CSK activity (*40*, *41*), we also assessed LCK Tyr^192^ phosphorylation in J.Csk^AS^/CD45 cells, which lack CD45 and can be subjected to inhibition of CSK-analog-sensitive (Csk^AS^) activity to treatment with the PP1 analog compound 3-IB-PP1 (*40*). LCK Tyr^192^ phosphorylation was strikingly induced by 3-IB-PP1 in J.Csk^AS^/CD45 cells (Fig. 5C), ruling out CSK as a major LCK Tyr^192^ kinase and showing that restoration of LCK activity (as indicated by enhanced ZAP70 Tyr^319^ and PLCϒ Tyr^783^ phosphorylation; Fig. 5C) is sufficient to enhance LCK Tyr^192^ phosphorylation in the absence of CD45.

We next reasoned that if LCK Tyr^192^ is an LCK autophosphorylation site and is dephosphorylated by CD45, the increased phosphorylation of Tyr^192^ observed in J.N22 449 cells could be due to a ZAP70-CD45 retrograde feedback loop that inhibits LCK Tyr^192^ phosphorylation in T cells. We explored the effects of ZAP70 on LCK Tyr^192^ phosphorylation in cells lacking CD45 by knocking down ZAP70 in J45.01 cells or CD45 in P116 cells. CD45 knockdown strongly increased LCK Tyr^192^ phosphorylation in P116 cells (Fig. 5D), while ZAP70 knockdown did not enhance LCK Tyr^192^ phosphorylation in J45.01 cells (Fig. 5E). We reasoned that decreased LCK Tyr^192^ phosphorylation in cells lacking PTPN22 might be due to constitutive activation of the retrograde ZAP70-CD45 feedback loop (see also the effect of CSK knockdown in Fig. 4F) and thus decided to assess CD45 recruitment to LCK by PLA assay in TCR-stimulated J.N22 KO, J.N22 WT and J.N22 449 cells. As shown in Fig. 4F, a substantial increase in the molecular colocalization of CD45 with LCK was observed in KO cells versus WT cells. In contrast, PTPN22 S449A induced a further decrease in the molecular colocalization of CD45 with LCK compared with WT cells after T cell activation (Fig. 5F). Together, these data support CD45 as a key LCK Tyr^192^ phosphatase and that ZAP70 promotes this function of CD45 downstream TCR engagement by inducing proximity between CD45 and LCK. Phosphorylation of PTPN22 Ser^449^ enhances such CD45-mediated negative regulation of LCK Tyr^192^ by selectively impairing PTPN22 recruitment to and dephosphorylation of ZAP70. Our data are consistent with the reported model that LCK Tyr^192^ works upstream LCK Tyr^505^ to inhibit LCK (*14*).

### PTPN22 Ser^449^ phosphorylation enhances T cell activation and modulates effector functions by promoting dephosphorylation of LCK Tyr^192^

Next, we sought to elucidate the role of LCK Tyr^192^ phosphorylation in the mechanism of action of phospho-Ser^449^ PTPN22. Through potentially deeper inhibition of early TCR signaling and/or modulation of the ratio of LCK/ZAP70 activation, the retrograde inhibition of LCK by selective ZAP70 dephosphorylation observed in J.N22 449 cells could result in qualitative differences in the pathways that are ultimately activated by TCR engagement. We decided to take a multiomic approach to this problem by expressing monomeric enhanced green fluorescent protein (mEGFP) WT or S449A PTPN22 in J.CaM 1.6 cells engineered to reexpress LCK WT (J.Lck WT) or LCK Y192F (J.Lck Y192F) (*14*), which we confirmed to be devoid of LCK-Tyr^192^ phosphorylation (fig. S5A), and assessing downstream signaling in these cells through cellular indexing of transcriptomes and epitopes sequencing (CITE-seq) by 3′ GEM (Gel Bead-in-EMulsion) single-cell sequencing (scRNA-seq, 10× Genomics). To assess the intensity of TCR signal transduction at the single-cell level, we added adapters conjugated with an antibody against the TCR-induced molecule CD69, and to allow quantification of the PTPN22 constructs via 3′ GEM scRNA-seq methods, 450–base pair (bp) nucleotides sequences at the C-terminal open reading frame were optimized via silent mutagenesis (table S3 and fig. S5A). We confirmed that mEGFP-tagged PTPN22 S449A retained a higher ability to inhibit the NFAT/AP-1 luciferase reporter activity compared with WT PTPN22 (fig. S5B).

**Fig. 6.**
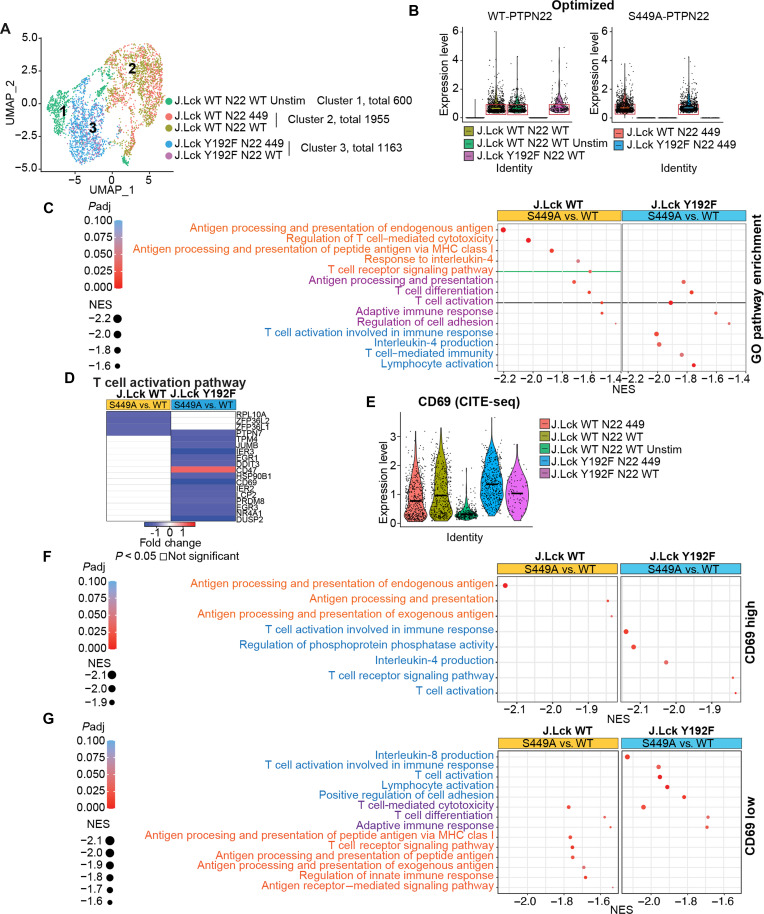
PTPN22 Ser^449^ phosphorylation enhances T cell activation and modulates effector functions by promoting dephosphorylation of LCK Tyr^192^. (**A**) Uniform Manifold Approximation and Projection (UMAP) of J.Lck cell clusters expressing optimized PTPN22 gene sequences. (**B**) Violin plots showing expression of optimized PTPN22 WT and S449A genes in single J.Lck WT or J.Lck Y192F cells; each dot represents a single cell; horizontal bars show median expression value. (**C**) Dot plot of GSEA GO pathway enrichment analysis of the differentially expressed genes (DEGs) in the optimized S449A versus WT populations of J.Lck WT and Y192F cells (*P*-adjust <0.05). NES, normalized enrichment score. (**D**) Heatmap of genes related to T cell activation enriched between optimized S449A and WT groups in J.Lck WT and Y192F cells (*P* < 0.05). (**E**) Violin plots showing the distribution of CD69 activation by CITE-seq; horizontal bars show median expression value. (**F**) Dot plot of GSEA GO pathway enrichment analysis of the DEGs in the optimized S449A versus WT populations of highly activated (as estimated by CD69 expression over median value of 0.65) J.Lck WT or Y192F cells. (**G**) Dot plot of GSEA GO pathway enrichment analysis of the DEGs in the optimized S449A versus WT populations of less activated J.Lck WT or Y192F cells (CD69 expression of all expressing cells below median 0.65). Specific enriched pathways are labeled in different colors (orange, purple, or blue).

We next subjected the four TCR-stimulated cell groups (J.Lck WT N22 WT, J.Lck WT N22 449, J.Lck Y192F N22 WT, and J.Lck Y192F N22 449), plus a group of unstimulated cells expressing WT LCK and PTPN22 (J.Lck WT N22 WT Unstim), to sorting for mEGFP^+^ cells followed by CITE-seq (fig. S5A). We selected cells expressing optimized PTPN22 mRNA sequences for analysis after sequencing, which revealed three distinct cell clusters (Fig. 6A) corresponding to J.Lck WT N22 WT Unstim (cluster 1, total 600), J.Lck WT (cluster 2, total 1955), and J.Lck Y192F (cluster 3, total 1163) cells. The distribution of optimized PTPN22 expression was comparable among the cell groups (Fig. 6B). Next, we split the cells expressing optimized PTPN22 at the median of PTPN22 expression and performed differential expression analysis with below the median cells, which showed more narrowly and homogeneously distributed PTPN22 expression (rectangles in Fig. 6B). Using these results, Gene Set Enrichment Analysis (GSEA) was performed with the Gene Ontology (GO) biological process database terms and normalized enrichment scores (NES) and significance values were calculated. Several pathways related to T cell responses were down-regulated (NES < −1.4, *P*-adj < 0.05) in cells expressing S449A PTPN22, which is consistent with the gain-of-function phenotype of PTPN22 S449A (Fig. 6C), but we noticed that pathways down-regulated in cells expressing WT versus Y192F LCK were only partially overlapping. Pathways regulating antigen processing and presentation, T cell differentiation, molecules involved in T cell activation, adaptive immune responses, and regulation of cell adhesion were enriched in all cells expressing S449A PTPN22 (purple rows in Fig. 6C). However, pathways related to antigen processing and presentation of endogenous antigen, regulation of T cell–mediated cytotoxicity, and response to IL-4 and TCR signaling were selectively enriched in cells expressing LCK WT (orange rows in Fig. 6C), while pathways related to T cell activation involved in immune responses, IL-4 production, T cell–mediated immunity, and lymphocyte activation were enriched in cells expressing the Y192F mutant (blue rows in Fig. 6C). Notably, even if the TCR activation gene pathway was inhibited in cells expressing either LCK WT or the Y192F mutant (black line, Fig. 6C), the pattern of genes whose expression was inhibited by S449A PTPN22 depended on LCK Tyr^192^ phosphorylation (Fig. 6D). To assess whether the functional interaction between PTPN22 Ser^449^ and LCK Tyr^192^ phosphorylation depends on the intensity of T cell activation (which in turn affects the stoichiometry of Ser^449^ phosphorylation), we leveraged the CD69 data obtained by CITE-seq. At the population level, CD69 expression was up-regulated in stimulated versus unstimulated cells (Fig. 6E); however, consistent with PTPN22 S449A causing inhibition of TCR signaling pathway only in the presence of WT Lck (green line, Fig. 6C), the expression of PTPN22 S449A correlated with lower expression of CD69 in J.Lck WT cells but not in J.Lck Y192F cells. In highly stimulated T cells (CD69 high, expression level over median value), LCK Tyr^192^ phosphorylation was required for S449A PTPN22 to down-regulate pathways involved in antigen processing and presentation (orange rows in Fig. 6F) but inhibited the down-regulation of pathways associated with T cell activation, regulation of phosphoprotein phosphatase activity, IL-4 production, and TCR signaling (blue rows in Fig. 6F). In less stimulated T cells (CD69 low, expression level below the median value), several pathways including T cell–mediated cytotoxicity, T cell differentiation, and adaptive immune response were down-regulated by S449A PTPN22 independent of LCK phosphorylation (purple rows in Fig. 6G). LCK Tyr^192^ phosphorylation was required for S449A PTPN22 to down-regulate pathways related to antigen processing, TCR signaling, innate immune response, and antigen receptor–mediated signaling (orange rows in Fig. 6G), and inhibited the down-regulation of pathways related to IL-8 production, T cell activation, lymphocyte activation, and cell adhesion caused by S449A PTPN22 (blue rows in Fig. 6G). Together, these data confirm the gain-of-function nature of S449A PTPN22. LCK Tyr^192^ dephosphorylation quantitatively and qualitatively modulates the promotion of early TCR signaling operated by PTPN22 Ser^449^ phosphorylation over the full range of TCR activation, and the outcome of such feedback interaction is influenced by the intensity of TCR stimulation.

### Phosphorylation of PTPN22 Ser^449^ facilitates T cell recognition of peptide antigens presented by MHC

**Fig. 7.**
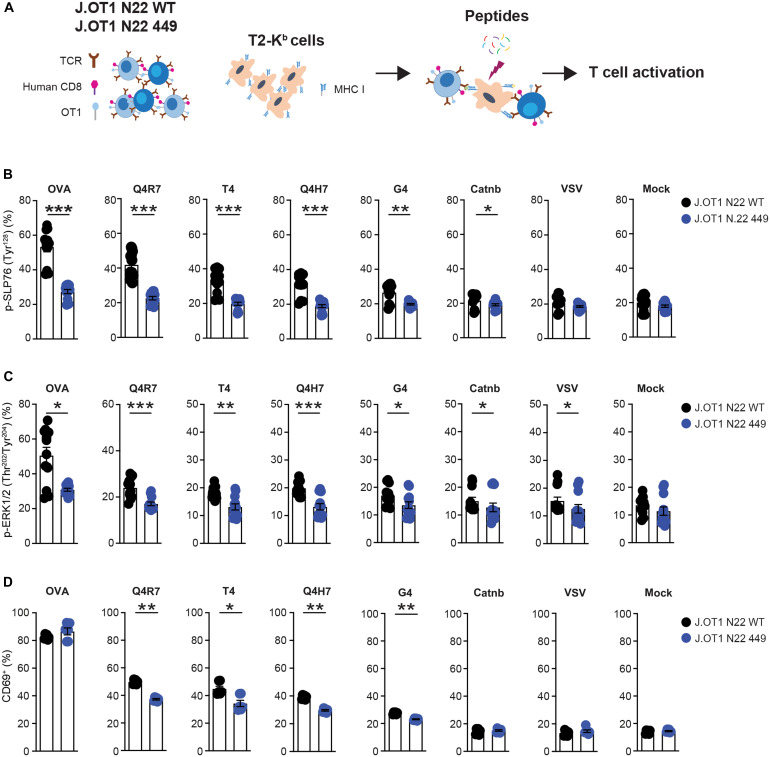
Phosphorylation of PTPN22 Ser^449^ facilitates T cell recognition of peptide antigens presented by MHC. (**A**) Schematic illustration of the interaction of J.OT1N22 and J.OT1N22 449 cells with antigen-presenting cells pulsed by multiple peptides. (**B** to **D**) Flow cytometry analysis of SLP-76, extracellular signal–regulated kinase (ERK) phosphorylation, and CD69 activation in J.OT1 N22 WT or J.OT1 N22 449 cells stimulated with or without 1 nM OVA, Q4R7, T4, Q4H7, G4, Catnb, or VSV control peptide–pulsed T2-K^b^ cells for the indicated time. Percentage of SLP-76 [Tyr^128^ (B)] and ERK1/2 [Thr^202/^Tyr^204^ (C)] phosphorylation was measured after stimulation for 1 and 5 min, respectively. The activation of CD69 (as % CD69^+^ cells) was assessed after 16 hours of stimulation (D). Data in histogram are presented as means ± SEM and are from at least three (B to D) independent experiments. Statistical significance was assessed by two-tailed Mann-Whitney test. *P < 0.05, **P < 0.01, ***P < 0.001. (A) created with BioRender (*69*).

PTPN22 is known to preferentially inhibit TCR responses to weaker ligands (*42*). To assess the phospho-Ser^449^–based PTPN22 regulation mechanism across a range of ligand strengths, we studied J.OT1.hCD8^+^ Jurkat cells (*43*) stimulated with TCR ligands of varying affinity presented by T2-K^b^ cells (*44*). We generated PTPN22 KO (J.OT1 N22 KO) and 3× FLAG tag KI PTPN22 (3× FLAG N22 J.OT1) J.OT1.hCD8^+^ Jurkat cells via CRISPR-Cas9 editing (fig. S6, A and B). Next, we assessed PTPN22 Ser^449^ phosphorylation in 3× FLAG N22 J.OT1 cells stimulated by T2-K^b^ cells pulsed with ovalbumin (OVA) peptide (residues 257 to 264) and found it dramatically increased at 30 min (fig. S6C). We then repeated the experiment in 3× FLAG N22 J.OT1 cells stimulated by T2-K^b^ cells pulsed with an array of varying affinity peptides [OVA, Q4R7, T4, Q4H7, G4, beta catenin (Catnb), and vesicular stomatitis virus (VSV)] for 30 min (fig. S6D). Ser^449^ phosphorylation was increasingly induced by higher affinity peptides (OVA, Q4R7, T4, and Q4H7), while lower affinity, self or control peptides (G4, Catnb, and VSV) induced similar lower levels of phosphorylation (fig. S6E). We next generated J.OT1 N22 449 (PTPN22 S449A) cells via CRISPR-Cas9 editing (fig. S6, F and G) and stimulated these cells with T2-K^b^ pulsed with the abovementioned OVA peptides (Fig. 7A). Phosphorylation of SLP-76 and extracellular signal–regulated kinase (ERK) were reduced in J.OT1 N22 449 cells with differences proportional to the affinity of the stimulating peptide and gradually growing from weak to strong peptides (Fig. 7, B and C, and fig. S6H). We observed a similar pattern of affinity-correlated and enhanced versus reduced CD69 induction in J.OT1 N22 WT versus J.OT1 N22 449 cells, respectively; however, differences were notably absent in cells stimulated with the strongest ligand OVA (Fig. 7D and fig. S6H). These data support the idea that PTPN22 Ser^449^ phosphorylation contributes to the ability of cells to discriminate among ligands over a wide range of affinities and potencies. High stoichiometry phosphorylation and inhibition of PTPN22 by strong ligands might be consistent with the reported preferential enhancement of weak ligand signaling in PTPN22 KO cells (*42*).

### Phosphorylation of Ptpn22 Ser^452^ promotes TCR signaling and adaptive responses to immunization

**Fig. 8.**
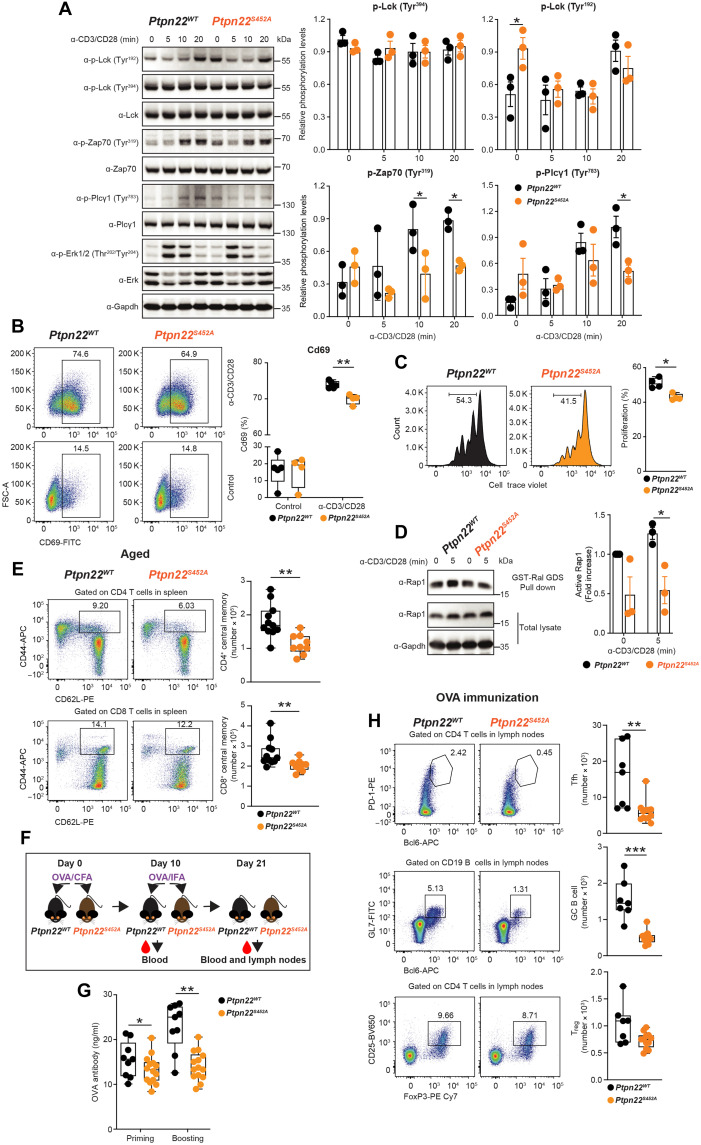
Phosphorylation of Ptpn22 Ser^452^ promotes TCR signaling and adaptive responses to immunization. (**A**) Protein phosphorylation induced by TCR stimulation in CD4^+^ effector T cells from *Ptpn22^WT^* (*n* = 3) or *Ptpn22^S452A^* mice (*n* = 3) analyzed by Western blotting (left) and quantification of phosphorylated Lck, Zap70, and Plcϒ normalized to total proteins (right). (**B**) Flow cytometry analysis of Cd69 activation in CD4^+^ effector T cells from *Ptpn22^WT^* (*n* = 4) or *Ptpn22^S452A^* (*n* = 4) mice stimulated with or without antibodies against mouse CD3/CD28. (**C**) Flow cytometry analysis of proliferation of CD4^+^ effector T cells from *Ptpn22^WT^* (*n* = 4) or *Ptpn22^S452A^* (*n* = 4) mice. (**D**) Endogenous Rap1 activity in CD4^+^ effector T cells from *Ptpn22^WT^* (*n* = 3) or *Ptpn22^S452A^* (*n* = 3) mice was assessed through Ral GDS pull down and detected by immunoblotting, and quantification of the precipitated Rap1 was normalized to WT at time 0. (**E**) Changes in the absolute numbers of CD4^+^ and CD8^+^ T cells with naïve (CD44^lo^ CD62L^hi^), effector (CD44^hi^ CD62L^lo^), and central memory (CD44^hi^ CD62L^hi^) phenotypes in spleens of aged (over 6 months) *Ptpn22^WT^* (*n* = 11) or *Ptpn22^S452A^* (*n* = 9) mice. (**F**) An illustration of OVA immunization of *Ptpn22^WT^* or *Ptpn22^S452A^* mice. (**G**) OVA-specific IgGs were quantified in 8-week *Ptpn22^WT^* (*n* = 9) or *Ptpn22^S452A^* (*n* = 14) mice immunized by subcutaneous injection at day 10 (priming) and 21 (boosting). (**H**) Numbers of Tfh (Tcrβ^+^ CD4^+^ PD-1^+^ Bcl6^+^), GC B cells (Tcrβ^−^ CD19^+^ GL7^+^ Bcl6^+^), and T_reg_ cells (Tcrβ^+^ CD4^+^ CD25^+^ FoxP3^+^) in draining lymph nodes of OVA-immunized *Ptpn22^WT^* (*n* = 7) or *Ptpn22^S452A^* (*n* = 12) mice were analyzed by flow cytometry at 21 days after primary immunization. Representative blots and flow gating are shown (A to D, E, and H). Data represent means ± SEM, and statistical significance was assessed by two-way ANOVA followed by Bonferroni’s post hoc test (A, B, and D) and two-tailed Mann-Whitney test (C, E, G, and H). *P < 0.05, **P < 0.01, ***P < 0.001.

Next, to determine whether phosphorylation on Ser^449^ (Ser^452^ in mouse Ptpn22) regulates the function of Ptpn22 in vivo, we generated a mouse model carrying a *Ptpn22^S452A^* mutation (fig. S7, A and B). We noticed that phosphorylation of Ptpn22 at Ser^452^ selectively decreases the binding efficiency of a monoclonal PTPN22 antibody raised against a PTPN22 peptide surrounding Pro^451^ (Cell Signaling Technology), while it does not affect the binding of a polyclonal Ptpn22 antibody raised against the P1 motif (amino acids 588 to 654) (fig. S7C, top) (*17*). Using these two antibodies, we could confirm that Ser^452^ phosphorylation was eliminated by the *Ptpn22^S452A^* mutation without alterations in Ptpn22 expression (fig. S7C, bottom). We then proceeded to assess TCR signaling in CD4^+^ effector T cells from homozygous *Ptpn22^S452A^* versus *Ptpn22^WT^* mice. Consistent with the observations in human cells, the phosphorylation of Zap70 Tyr^319^ and its downstream substrate Plcϒ Tyr^783^ in CD4^+^ effector T cells of *Ptpn22^S452A^* mice were inhibited compared to cells from *Ptpn22*^WT^ mice (Fig. 8A), while phosphorylation of Lck Tyr^394^ was unaffected. Lck Tyr^192^ phosphorylation was up-regulated in *Ptpn22^S452A^* mice at time 0 and then down-regulated by TCR stimulation (Fig. 8A). TCR-induced Cd69 expression in the *Ptpn22*^S452A^ mice was notably inhibited after 4 hours of stimulation (Fig. 8B). PTPN22 is known to inhibit TCR-induced T cell proliferation (*17*); thus, we further evaluated proliferation of CD4^+^ effector T cells from *Ptpn22^WT^* or *Ptpn22^S452A^* mice by CellTrace Violet staining. As shown in Fig. 8C, TCR-induced CD4^+^ T cell proliferation was considerably reduced in *Ptpn22^S452A^* mice. Consistent with the finding in Jurkat cells (Fig. 2G), we also found Rap1 activity to be reduced in CD4^+^ effector T cells from *Ptpn22*^*S452A*^ mice when compared to *Ptpn22^WT^* T cells after TCR stimulation (Fig. 8D). In response to stimulation with OVA peptide (residues 329 to 336, specific for OT-II^+^ TCR) pulsed splenocytes, OT-II^+^ CD4^+^ T cells from *Ptpn22*^*S452A*^ mice displayed blunted induction of Cd69, and a similar Ptpn22 gain-of-function phenotype was also observed when T cells from OT-II^+^ CD4^+^ T cells from *Ptpn22^S452A^* mice were stimulated with E336Q peptide (a partial agonist of OVA peptide) pulsed splenocytes. In these assays, cells stimulated with self-peptide Class II-associated invariant chain peptide (CLIP) pulsed splenocytes did not induce any Cd69 activation (fig. S7, D to F).

Increased positive thymic selection has been reported in Ptpn22 KO mice (*17*), and Tyr^192^ phosphorylation impairs thymic reconstitution of retrogenic mice (*14*). However, when we analyzed the thymus of 4-week-old *Ptpn22^WT^* or *Ptpn22^S452A^* mice, we did not find alterations of double-negative, CD4 or CD8 single-positive, or double-positive thymocyte populations in *Ptpn22^S452A^* mice (fig. S8A). KO of Ptpn22 in mice enhances responses to immunization and leads to a marked expansion of effector memory CD4 and CD8 T cells in aged (>6 months old) animals (*16*, *17*). We immunophenotyped T cells in spleen and lymph nodes of >6-month-old *Ptpn22^S452A^* versus littermate WT mice and found that aged *Ptpn22^S452A^* mice displayed reduced numbers of CD4^+^ and CD8^+^ central memory T cells in spleens (Fig. 8E), a phenotype that was not evident in lymph nodes (fig. S8, B and C). Next, we immunized *Ptpn22^WT^* or *Ptpn22^S452A^* mice with OVA (Fig. 8F) and observed decreased titers of anti-OVA immunoglobulin G (IgG) antibodies in *Ptpn22^S452A^* mice after both priming and boosting injections (Fig. 8G). Immunophenotyping of draining lymph nodes after boosting showed that CD4^+^ T follicular helper (Tfh) and germinal center (GC) B cell frequencies were highly reduced in the draining lymph nodes of *Ptpn22^S452A^* mice, while there was no change in regulatory T cell (T_reg_) numbers (Fig. 8H and fig. S8D). Together, these results support the notion that Ptpn22 phospho-Ser^452^ reduces the inhibitory function of Ptpn22 on TCR signaling in vivo.

### Ptpn22 Ser^452^ phosphorylation promotes disease severity in a T cell–dependent lupus mouse model

**Fig. 9.**
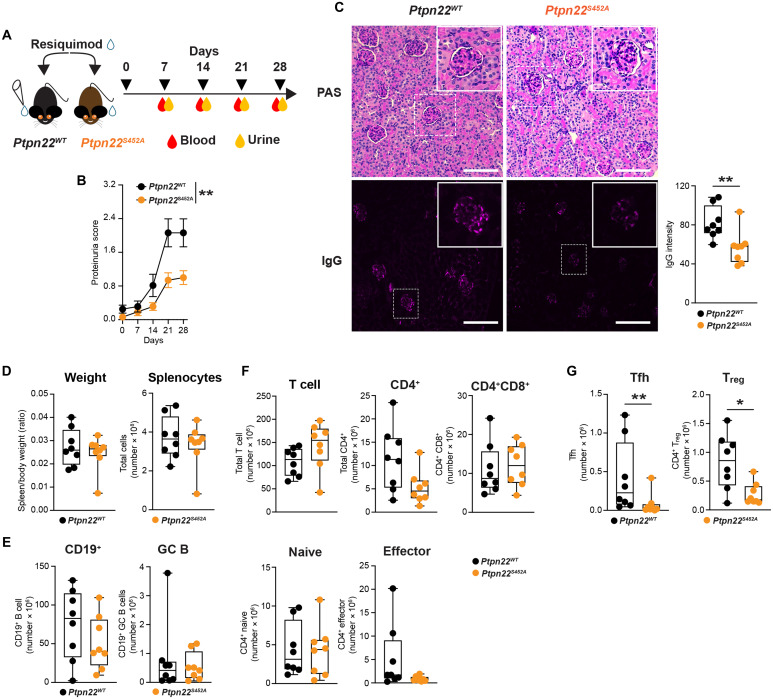
Ptpn22 Ser^452^ phosphorylation promotes disease severity in a T cell–dependent lupus mouse model. (**A**) Schematic illustration of the 28-day R848 treatment protocol performed on *Ptpn22^WT^* (*n* = 8) or *Ptpn22^S452A^* (*n* = 8) mice. (**B**) Proteinuria score of *Ptpn22^WT^* or *Ptpn22^S452A^* mice treated with R848 for 28 days and measured every 7 days. (**C**) Periodic acid–Schiff (PAS) staining of kidney sections and immunohistochemical staining of IgGs in kidney glomeruli from R848-treated *Ptpn22^WT^* or *Ptpn22^S452A^* mice. Images were taken at 10× magnifications. Scale bars, 50 μm. IgG intensity in the glomeruli was quantified in each mouse (right). (**D**) Spleen/body weight ratio and total splenocytes comparison between *Ptpn22^WT^* and *Ptpn22^S452A^* R848-treated mice. (**E** to **G**) Splenocytes analysis by flow cytometry was performed on cells collected at day 28 from *Ptpn22^WT^* or *Ptpn22^S452A^* mice, and total numbers of target cell populations were quantified. Numbers of total B cells (Tcrβ^−^ CD19^+^), GC B cells (Tcrβ^−^ CD19^+^ GL7^+^ Bcl6^+^) (E); total T cells (Tcrβ^+^), CD4^+^ T cells (Tcrβ^+^ CD4^+^), CD4^+^ CD8^+^ T cells (Tcrβ^+^ CD4^+^CD8^+^), CD4^+^ naïve (Tcrβ^+^ CD4^+^CD44^lo^ CD62L^hi^), CD4^+^ effector (Tcrβ^+^ CD4^+^CD44^hi^ CD62L^lo^) T cells (F), Tfh (Tcrβ^+^ CD4^+^ PD-1^+^ Bcl6^+^), and T_reg_ cells (Tcrβ^+^ CD4^+^ CD25^+^ FoxP3^+^) (G) were quantified. Data are presented as means ± SEM, and statistical significance was assessed by two-way ANOVA followed by Bonferroni’s post hoc test (B) and two-tailed Mann-Whitney test (C to G). *P < 0.05, **P < 0.01.

Given that PTPN22 phospho-Ser^449^ functionally regulates PTPN22 to promote T cell responses and PTPN22 has a strong genetic association with SLE, we next sought to collect data to support a role of Ser^449^ phosphorylation in SLE pathogenesis by subjecting *Ptpn22^S452A^* mice to a T cell–dependent inducible model of SLE (*45*, *46*). Repeated topical treatment of C57BL/6 mice with resiquimod (R848) induces T cell activation, splenomegaly, B cell expansion with hypergammaglobulinemia, IgG deposition in kidney, and overt proteinuria (see outline in Fig. 9A). When subjected to the R848 lupus model, *Ptpn22^S452A^* mice displayed markedly attenuated proteinuria level (Fig. 9B) correlating with reduced deposition of IgG in kidney glomeruli, pointing to protection against lupus nephritis (Fig. 9C). The *Ptpn22^S452A^* mutation did not attenuate R848-induced splenomegaly (Fig. 9D). Immunophenotyping of the spleen showed comparable numbers of splenocytes in *Ptpn22^S452A^* versus *Ptpn22^WT^* mice (Fig. 9D), and there were no changes in the total number of B cells (Tcrβ^−^ CD19^+^) or GC B cells (Tcrβ^−^ CD19^+^ GL7^+^ Bcl6^+^) (Fig. 9E and fig. S8E). Numbers of total splenic T cells, CD4^+^ (Tcrβ^+^ CD4^+^) T cells, CD4^+^ naïve (CD44^lo^ CD62L^hi^) T cells, and CD4^+^ effector (CD44^hi^ CD62L^lo^) T cells were also comparable between *Ptpn22^S452A^* versus *Ptpn22^WT^* mice (Fig. 9F and fig. S8F); however, Tfh (Tcrβ^+^ CD4^+^ PD-1^+^ Bcl6^+^) and T_reg_ cells (Tcrβ^+^ CD4^+^ CD25^+^ Foxp3^+^) were strongly reduced in the spleen of *Ptpn22^S452A^* mice (Fig. 9G and fig. S8G). Together, these data suggest that Ptpn22 Ser^452^ phosphorylation enhances T cell responses to promote SLE pathogenesis.

## DISCUSSION

PTPN22, a key negative regulator of TCR signaling, is encoded by a gene whose R620W variation is strongly associated with SLE (*47*, *48*). Here, through phosphoproteomics analysis, we identified Ser^449^ as a major TCR-induced PKA phosphorylation site of PTPN22 in T cells. PKA contributes to modulating signaling downstream TCR engagement in response to a variety of concurrent stimuli (*49*), and we show that PKA activity is induced in T cells by early TCR engagement. We find that PKA Cα can directly phosphorylate PTPN22 on Ser^449^ and is required for TCR-induced PTPN22 phosphorylation. We also show that PBMCs from patients with SLE and active disease display elevated Ser^449^ phosphorylation. Our data about the function of Ser^449^ phosphorylation in TCR signaling and the phenotype of *Ptpn22^S452A^* mice suggest that the hyperphosphorylation of Ser^449^ found in SLE might contribute to the pathogenesis of T cell hyperactivation reported in SLE and in disease mechanism (*50*). The protective effect of gain-of-function Ptpn22^S452A^ against experimental lupus is also consistent with various reports suggesting that the lupus-associated PTPN22-R620W variation alters or reduces the function of PTPN22 (*16*). The hyperphosphorylation of PTPN22 observed in lupus PBMCs could be secondary to the intrinsic abnormalities of PKAs and alterations of the TCR signaling complex that have been reported in SLE T cells (*2*, *28*). However, cytokine signaling dysregulation due to disease activity could also play a role and, for example, IFN-γ can activate PKA signaling and is increased in active SLE (*51*, *52*). Further studies are needed to fully clarify regulation of PTPN22 Ser^449^ by PKA in healthy and lupus cells, whether the hyperphosphorylation of Ser^449^ is limited to lupus T cells and its relationship with disease severity in SLE.

To explore effects of PTPN22 Ser^449^ phosphorylation on TCR signaling, we generated a PTPN22 S449A Jurkat KI line by CRISPR-Cas9–mediated genome editing. Analysis of these cells compared to cells carrying WT PTPN22 showed a reduction in TCR-induced phosphorylation of activating residues of proximal TCR signaling mediators ZAP70, SLP-76, and PLCϒ, and reduced expression of CD69, IL-2, and TNF-α, suggesting that PTPN22 Ser^449^ phosphorylation enhances early TCR signaling and T cell activation. To more deeply characterize the role of PTPN22 Ser^449^ phosphorylation in regulation of T cell function, we also generated a KI mouse model carrying the Ptpn22 S452A mutation. Concordant with the effect of Ser^449^ mutation in human T cells, loss of this phosphorylation site in mice led to reduced TCR signaling, decreased numbers of CD4^+^ and CD8^+^ central memory T cells in spleens of aged mice, and reduced antigen-specific IgG antibodies following priming and boost immunizations, suggesting diminished peripheral T cell responses. These phenotypes are mostly opposite to the ones reported for mice carrying deletion or R619W mutation of Ptpn22, with the exception of thymocyte counts that were not affected in Ptpn22 S452A mice. The latter could be due to potential repertoire-based or other compensation mechanisms in the context of PTPN22 gain-of-function and is consistent with the previously reported phenotype of mice overexpressing human PTPN22 (*53*). *Ptpn22^S452A^* mice also showed decreased disease progression in the R848-induced model of lupus when compared to WT mice. The splenic T cell phenotypes from this lupus model, combined with the abovementioned immunophenotype of healthy *Ptpn22^S452A^* mice suggest that reduced TCR signaling underlies at least in part the observed protection from disease. However, our KI mouse model causes a global S452A mutation of PTPN22, thus a contribution of other immune cells cannot be ruled out at the moment. As Ptpn22 also regulates IFN-α signaling (*54*), additional studies are also warranted to clarify whether Ser^449^ affects additional signaling pathways that are relevant to the pathogenesis of lupus in humans and in R848-induced lupus-like disease.

We observed increased phosphorylation of PTPN22 Ser^449^ in J.OT1 cells stimulated with higher affinity OVA-derived peptide ligands, suggesting that induction of phosphorylation at this site is dependent on the strength of TCR signaling. This may in part explain the observed greater effect of PTPN22 Ser^449^ phosphorylation on OVA-derived peptide ligand–induced phosphorylation of early TCR signaling mediators. In mouse OT-II–expressing CD4^+^ T cells, Ptpn22 S452A suppressed signaling to a greater extent than WT Ptpn22 when cells were stimulated with full-strength OVA peptide, while in human OT-I Jurkat T cells, there was no effect of PTPN22 S449A on TCR signaling in response to OVA peptide. This may be due to a higher affinity of OVA for OT-I versus OT-II TCRs, causing a greater strength of stimulation that overcame the effect of PTPN22 phosphorylation in OVA-stimulated OT-I cells. Thus, in vivo Ser^449^ phosphorylation might contribute to the reported role of PTPN22 in discriminating strong versus weak agonists and enhancing signaling induced by stimuli of weak and intermediate strength.

Since PTPN22 S449A cells exhibit impaired TCR signaling and downstream T cell activation, we initially assumed that phosphorylation on this site directly inhibited the catalytic activity of PTPN22. However, we had noted that S449A cells display equivalent TCR-induced phosphorylation of LCK Tyr^394^ and PTPN22 immunoprecipitated from TCR-stimulated S449A cells showed equivalent phosphatase activity to PTPN22 immunoprecipitated from WT cells, suggesting that phosphorylation affects the ability of PTPN22 to bind and/or dephosphorylate ZAP70. Compared to WT PTPN22, PTPN22 S449A showed enhanced colocalization in TCR-stimulated cells with ZAP70 but not LCK. Furthermore, in intracellular dephosphorylation assays in COS-7 cells, expression of PTPN22 S449A had the same effect as WT PTPN22 on the phosphorylation of coexpressed LCK Tyr^394^ yet led to reduced phosphorylation of coexpressed ZAP70 on Tyr^319^ and Tyr^493^. These results are likely due to a conformational change in phosphorylated PTPN22, as PTPN22 S449A showed enhanced FRET compared to WT PTPN22 when fused at the N and C termini to cyan fluorescent protein and yellow fluorescent protein, respectively. The location of the Ser^449^ phosphorylation site within the PTPN22 interdomain may facilitate an altered PTPN22 structure upon phosphorylation that lowers the affinity for ZAP70 while keeping the structural determinants needed for LCK binding intact. We do not exclude the possibility that Ser^449^ phosphorylation operates through a more indirect mechanism or affects recognition of other PTPN22 substrates.

In our examinations of the effect of PTPN22 phosphorylation on LCK activation state, we uncovered the unexpected finding that LCK Tyr^192^ is more phosphorylated in T cells where PTPN22 Ser^449^ phosphorylation is abolished. Although LCK Tyr^192^ has been reported to be phosphorylated by ITK (*15*), our data suggest that in our T cell system, LCK Tyr^192^ is primarily an autophosphorylation site, as Tyr^192^ phosphorylation is unaffected by cell treatment with an ITK inhibitor, reduced on the inactive Y394F LCK mutant, and strongly enhanced on the hyperactive Y505F LCK mutant. However, it is possible that phosphorylation of Tyr^192^ is under ITK control in specific T cell populations or conditions and that a similar TCR-driven retrograde loop could be promoted by an LCK-ITK mechanism rather than LCK alone in different contexts. Our data also point to a role for both ZAP70 and CD45 in the negative regulation of LCK Tyr^192^ phosphorylation state. Knockout or knockdown of ZAP70 and knockdown of CD45, enhances Tyr^192^ phosphorylation. Cells carrying PTPN22 S449A also showed reduced colocalization of CD45 with LCK. Together, these data suggest a model by which during TCR stimulation, LCK Tyr^192^ is autophosphorylated, which would depress TCR signaling. However, CD45 acts as a key negative regulator of LCK Tyr^192^ phosphorylation—possibly through direct dephosphorylation—through a mechanism that is enhanced by ZAP70 activation. Phosphorylation of PTPN22 Ser^449^ impairs its ZAP70 dephosphorylation ability, which results in enhanced CD45-mediated inhibition of LCK Tyr^192^ phosphorylation, and promotes strength of TCR signaling and T cell activation in a retrograde positive feedback loop.

A question that remains open is how the enhanced activation of ZAP70 upon PTPN22 Ser^449^ phosphorylation leads to increased dephosphorylation of LCK Tyr^192^ by CD45. We demonstrate through PLA that CD45 colocalization with LCK is regulated by PTPN22 Ser^449^ phosphorylation, suggesting that ZAP70 activation induces recruitment of CD45 to LCK. Whether this occurs through direct phosphorylation of CD45, CD45 complexing with phosphorylated adaptor proteins such as lymphocyte phosphatase–associated phosphoprotein (*55*) and/or other mechanisms remains to be clarified.

Our proposed retrograde feedback mechanism prompts the question of how relevant is the dephosphorylation of an inhibitory site on LCK induced by the activation of its downstream effector. The retrograde loop likely underlies the complete absence of phosphorylation of LCK Tyr^192^ in PTPN22 KO cells. However, interpreting the interaction between LCK Y192F and PTPN22 KO would be complicated by the considerable LCK hyperactivation caused by PTPN22 KO, which might obliterate the modulatory role of the CD45-mediated loop. We thus opted to study the interaction between LCK Y192F and PTPN22 S449A at the single-cell level using CITE-seq of cells expressing combination of mutants. The results of our analysis support the gain-of-function nature of S449A and previous data about the role of LCK Tyr^192^ in TCR signaling (*14*). They also suggest that LCK Tyr^192^ phosphorylation not only is important to sustain PTPN22 Ser^449^–phosphorylation-mediated promotion of TCR signaling but also modulate its outcome in terms of which pathways are preferentially promoted by Ser^449^ phosphorylation. We also show that, as expected, such differences are further modulated by the strength of TCR signaling, likely in part through control of Ser^449^ phosphorylation stoichiometry. Further studies are needed to understand whether such qualitative differences in signaling induced by the cross-talk between LCK Tyr^192^ and PTPN22 Ser^449^ phosphorylation have physiological relevance and whether they depend on overall intensity of TCR signaling downstream ZAP70 or the ratio of LCK/ZAP70 activation plays a role.

We believe that our data are sufficient to conclude that TCR-induced autophosphorylation of LCK Tyr^192^ balanced by PTPN22 phospho-Ser^499^–promoted dephosphorylation of the same site by CD45 allows for fine feedback regulation of LCK versus ZAP70 activation in response to stimuli of various strength, as well as a variety of additional variables. Our study also highlights the “linchpin role” of CD45 in early TCR signaling as the key phosphatase that regulates all three major phosphorylation sites of LCK in response to various cues. The opposite phenotypes we observed in CD45 KO versus knock-down cells is consistent with previous studies highlighting that CD45 complex function in TCR signaling can hardly be recapitulated by studies in cells completely lacking CD45 (*40*).

**Fig. 10.**
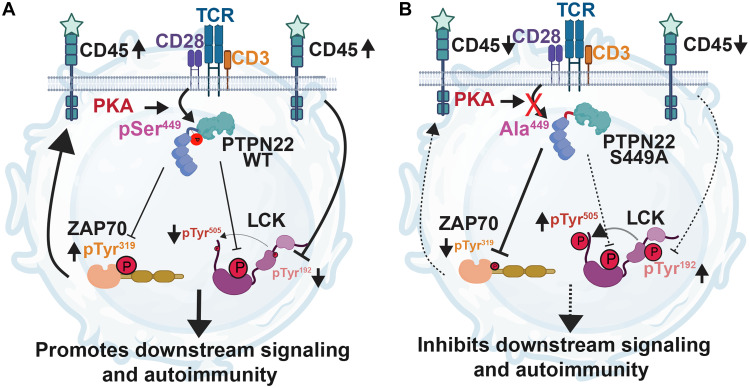
Current working model. (**A**) PTPN22 Ser^449^ is phosphorylated by PKA and induces a PTPN22 conformational change that selectively lowers the affinity of the phosphatase for phosphorylated ZAP70. The consequent hyperphosphorylation of ZAP70 promotes a ZAP70-CD45 feedback loop, which leads to dephosphorylation of LCK Tyr^192^, which has been shown to facilitate dephosphorylation of inhibitory Tyr^505^. The overall effect of PTPN22 phospho-Ser^449^ is to promote TCR signaling and autoimmunity, and it is abolished by the S449A mutation (**B**), which causes gain-of-function inhibition of TCR signaling and protects mice from autoimmunity. Schematic created with BioRender (*69*).

In summary, through a combination of phosphoproteomic, cell-based, in vivo, and multiomic approaches, we report Ser^449^ phosphorylation of PTPN22 as a key mechanism by which T cells enhance and modulate the outcome of TCR-induced signals through activation of a ZAP70-CD45-LCK signaling loop. Our model (Fig. 10, A and B) is that PKA-mediated phosphorylation of PTPN22 on Ser^449^ selectively inhibits PTPN22 binding to and dephosphorylation of ZAP70 on Tyr^319^ and Tyr^493^, resulting in dephosphorylation of LCK Tyr^192^ by CD45 and enhanced activation T cells. PTPN22 Ser^449^ phosphorylation contributes to support proximal TCR signaling, peripheral T cell activation, and promotion of SLE pathogenesis.

In addition to PTPN22, several other protein phosphatases including PP2A (*56*) and DUSP22 (*57*) have recently emerged as potential pathogenic players in SLE T cells. Further investigation into the regulation of PTPN22 and these other phosphatases in T cell is warranted to understand the complex intricacies of how T cells are activated and how TCR signaling pathways can be exploited to develop targeted immunotherapies for autoimmune diseases.

## MATERIALS AND METHODS

### Cells

Human PBMCs were isolated from peripheral blood [obtained from La Jolla Institute for Immunology's in-house Normal Blood Donor Program (NBDP), with EDTA] using SepMate tubes with Lymphoprep (STEMCELL Technologies) according to the manufacturer’s instructions. The human CD4^+^ T cells were enriched using the EasySep Human CD4^+^ T Cell Isolation Kit (STEMCELL Technologies) and expanded using Dynabeads Human T-Activator CD3/CD28 (Thermo Fisher Scientific) in the presence of recombinant human IL-2 (20 ng/ml; R&D Systems) for 5 days. J.OT1.hCD8^+^ cells, J.Csk^AS^/CD45, J.Lck WT, and J.Lck Y192F were gifts from A. Weiss (University of California, San Francisco) (*14*, *40*, *58*). These and the other Jurkat-derived cell lines were maintained in RPMI 1640 medium supplemented with 10% fetal bovine serum (FBS), 1 mM sodium pyruvate, 2 mM l-glutamine, d-glucose (2.5 mg/ml), 10 mM Hepes, penicillin (100 units/ml), and streptomycin (100 μg/ml). All the Jurkat and its derivative cell lines are summarized in table S4. Mouse CD4^+^ T cells were isolated from pooled spleens and lymph nodes by EasySep Mouse CD4^+^ T cell isolation kit (STEMCELL Technologies) and cultured with Dynabeads mouse T-Activator CD3/CD28 (Thermo Fisher Scientific) in complete RPMI 1640 medium supplemented with recombinant mouse Il-2 (10 ng/ml) for 3 days. HEK293T and COS-7 cell lines were purchased from American Type Culture Collection and cultured in Dulbecco’s modified Eagle’s medium supplemented with 10% FBS. All cells were kept under 5% CO_2_ at 37°C.

### Mice

Mice were maintained in a specific pathogen–free facility, and all experiments were carried out in accordance with the Institutional Animal Care and Use Committee–approved protocols at University of California, San Diego (no. S16098 and no. S17160) or Cedars Sinai Medical Center (CSMC, No. 010724). *Ptpn22*^*S452A*^ KI mice on the C57BL/6 genetic background were generated using CRISPR-Cas9 technology at the Transgenic Mouse Model Core Facility of the National Core Facility for Biopharmaceuticals, National Science and Technology Council, and the Animal Resources Laboratory of National Taiwan University Centers of Genomic and Precision Medicine (Taiwan). Briefly, the single guide RNA (sgRNA) sequence with a protospacer adjacent motif (PAM) site (NGG) (5′-ctctgctgaatcagttcaaa-3′) was selected following the CRISPick (*59*) and the Cas-OFFinder online resources (*60*). The sequence for the repairing template (including 5′ and 3′ homologous sequences and the codon of the S452A mutation -TCA > GCT, serine to alanine) was 5′-tcgtgtcctagtgctctgcccataaacacagcggacaggtatcacaattcaaaggggccggtaaaacggaccaaa***gct***actcccttcgaattgattcagcagagaaaaacaaatgacttggccgtgg-3′, (GCT was bold italicized), including an artificially inserted sequence for the *BstB*I restriction enzyme site (TTCGAA) and the synonymously mutated TTG codon for leucine at amino acid residue 457 (CTG > TTG) to facilitate recombination efficiency and future genotyping (Integrated DNA Technologies Inc.). The sgRNA and the recombinant Cas9 protein were purchased from Synthego Corporation. Electroporation was performed with premixed sgRNA and Cas9 added to the repairing DNA oligonucleotide template and fertilized eggs from C57BL/6J mice, which were then electroporated on a BEX electroporation apparatus (CUY21EDIT2) set at pulse three times; current, 165 mA; voltage, 30 V; and resistance, 160 kilohm. The primers for genotyping polymerase chain reaction (PCR) amplification and *Bst*BI digestion are as follows: VF1: 5′-ATGTGAAAACGACAAACCAGCA-3′, VR1: 5′-CAGCACTATTCGGAGGGGATGA-3′. To minimize off-target effects, the *Ptpn22*^*S452A*^ mice were backcrossed with C57BL/6J mice for six generations before being used for experimentation. OT II mice [JAX 004194, B6.Cg-Tg(TcraTcrb)425Cbn/J] and RAG2 KO mice (JAX 008449, B6.Cg-Rag2tm1.1Cgn/J) were obtained from the Jackson Laboratory. Genotyping of mice was performed by Transnetyx (Cordova, TN).

### Patient samples

All patients with SLE (*n* = 6) [as per the American College of Rheumatology diagnostic criteria (*61*)] and healthy controls (table S1) were recruited at CSMC. All participants provided informed written consent, and the study was approved in advance by the CSMC institutional review board (protocol no. 19627). SLEDAI scores ranged from 2 to 9 at the time of enrollment.

### Plasmids, antibodies, reagents and primers

C-terminal triple FLAG tagged (3× FLAG) full-length PTPN22 WT plasmid has been described (*24*) and its derived mutants were constructed by site-directed mutagenesis or overlap extension PCR using the primers in table S2. Custom-made PTPN22 FRET, mEGFP-tagged codon-optimized WT or S449A PTPN22 plasmids were purchased from GenScript (Piscataway, NJ). pD1321-AP WT Cas9 plasmid with red fluorescent protein (RFP) selection marker containing the guide RNA (gRNA) sequences was purchased from DNA 2.0 (ATUM). NFAT/AP-1 firefly luciferase and *Renilla* luciferase reporters were purchased from Promega (Madison, WI). All plasmids were confirmed by DNA sequencing.

The polyclonal rabbit anti-human phospho-PTPN22 Ser^449^–specific antibody was custom-made by Pacific Immunology (Ramona, CA). Rabbit anti-mouse Ptpn22 P1 domain antibody was from Genentech (San Francisco, CA). Goat anti-human PTPN22 polyclonal antibody (AF3428) was purchased from R&D systems (Minneapolis, MN). Rabbit anti-PTPN22 (D6D1H), anti–phospho-Src family (Tyr^416^) (D49G4), anti-LCK (73A5, D88), anti–phospho-ZAP70 (Tyr^493^) (2704), anti–phospho-ZAP70 (Tyr^319^) (65E4), anti-ZAP70 (D1C10E), anti–phospho–PLC-γ (Tyr^783^) (D6M9S), anti–PLC-γ (D9H10), anti–phospho-CREB (Ser^133^) (87G3), anti-CREB (48H2), anti-PKA Cα (4782), anti-CSK (C74C1), and anti–glyceraldehyde-3-phosphate dehydrogenase (D16H11) antibodies were from Cell Signaling Technology (Boston, MA). Mouse anti-FLAG M2 (F3165) and anti–β Actin (AC15) antibodies were purchased from Millipore Sigma (Burlington, MA). Rabbit anti-p56lck/LCK (phospho-Tyr^192^) (LS-C199194) antibody was from Lifespan Biosciences. Ultra-LEAF purified human CD3 antibody (clone, OKT3), Ultra-LEAF purified human CD28 antibody (clone, CD28.2), Ultra-LEAF purified anti-mouse CD3ε (clone, 145-2C11), and Ultra-LEAF purified anti-mouse CD28 (clone, 37.51) were purchased from BioLegend (San Diego, CA). The polyclonal goat anti-mouse Ig (553998) and mouse anti-Syrian/Armenian Hamster IgG (550637) used for cross-linking were from BD Biosciences (Carlsbad, CA). Horseradish peroxidase–conjugated anti-rabbit IgG (95017-556), anti-mouse IgG (76835-688), and anti-goat IgG (103262-184) were purchased from VWR (Radnor, PA).

Anti-FLAG M2 magnetic beads, dimethyl sulfoxide (DMSO), β-mercaptoethanol and recombinant human PKA Cα protein were purchased from Millipore Sigma (Burlington, MA). Pierce protease inhibitor tablets (EDTA-free), Dynabeads human or mouse T-Activator CD3/CD28 magnetic beads, DiFMUP, SuperScript III First-Strand Synthesis SuperMix, Platinum PCR SuperMix, and Phos-tag Acrylamide were purchased from Thermo Fisher Scientific (Rockford, IL). 2× Laemmli sample buffer was from Bio-Rad (Hercules, CA). Protein G Sepharose 4 Fast Flow was purchased from GE Healthcare (Chicago, IL). The dual-luciferase reporter assay system was purchased from Promega (Madison, WI). Endofree plasmid Maxi kit, QIAquick Gel Extraction kit, RNeasy Plus Micro Kit, and DNeasy Blood & Tissue kit were purchased from QIAGEN (Redwood City, CA). Mouse OVA sIgG (OVA-specific IgG) enzyme-linked immunosorbent assay kit was purchased from MyBioSource (San Diego, CA). OVA and relative mutated or control peptides were ordered or synthesized from GenScript (Piscataway, NJ). Human PKA, ZAP70, CSK, CD45, and nontargeted siRNA were purchased from Horizon Discovery (Lafayette, CO). Recombinant human or mouse cytokines were from R&D systems (Minneapolis, MN). All primers used in the study were synthesized by Integrated DNA Technologies.

### Kinase prediction

Kinase prediction was performed using NetPhos 3.1 software, with a prediction score of 0.5 or higher indicating a positive hit (*62*, *63*).

### Inhibitors and cell treatment

PKA (H89 2HCl), PKC (Sotrastaurin, GÖ 6983, Staurosporine, Ro 31-8220), RSK (LJH685), and ITK (BMS-509744) inhibitors were from SelleckChem (Houston, TX). For cell treatments, the cells were pretreated with the inhibitors for 30 min at 37°C and then stimulated with or without antibodies (1 μg/ml) against human CD3/CD28 in the presence of inhibitors or DMSO for the indicated times.

### CRISPR-Cas9 strategies for generation of PTPN22 S449A KI cell line

The 3× FLAG KI PTPN22 or PTPN22 KO Jurkat cell lines have been described (*24*). The 3× FLAG KI PTPN22 or PTPN22 KO in J.OT1.hCD8^+^ cells were constructed as previously described (*24*). These were used as a parent line to generate PTPN22 S449A KI Jurkat or J.OT1.hCD8^+^ cells as follows: gRNA targeting PTPN22 genomic DNA nucleotides sequences around Ser^449^ in Exon 13 was designed and cloned into pD1321-AP WT Cas9 plasmid. Plasmids containing about 834-bp homology-directed repair (HDR) arms with S449A mutation were ordered from GenScript (Piscataway, NJ). The HDR fragments were amplified by PCR using Platimum PCR SuperMix and purified by QIAquick Gel Extraction kit. The double-stranded DNA fragments were concentrated and quantitated by Nanodrop. Ten micrograms of Cas9 plasmid and 10 μg of HDR fragments carrying the S449A mutation were electroporated into WT or J.OT1 WT Jurkat cells for 24 hours. RFP^+^ cells were sorted into 96-well round-bottom plates using a SONY SH800 cell sorter; genomic DNA was extracted by DNeasy Blood& Tissue kit and used as template to screen positive clones by allele-specific PCR amplification. The mutation on mRNA was further confirmed by sequencing DNA synthesized by SuperScript III First-Strand Synthesis SuperMix using RNA extracted from positive candidate clones (RNeasy Plus Micro Kit) as template. The primers for HDR arms sequences amplification, positive candidates screening (allele specific primers), and *Csp*CI digestion were summarized in table S2.

### In vitro phosphorylation and phosphatase activity assays

In vitro kinase phosphorylation assay was performed as previously described (*24*). For phosphatase activity assays, PTPN22 from lysates of KO, WT, or S449A cells was immobilized on anti-FLAG M2 magnetic beads. Beads bound to target proteins were suspended in kinetics buffer [50 mM tris (pH 7.2), 1 mM dithiothreitol, and 0.01% Triton X-100], and combined with an equal volume of 1 mM DiFMUP dissolved in kinetics buffer containing 8% DMSO in round-bottom black 96-well polystyrene microplates (Corning). Phosphatase activity was then measured by continuously monitoring the fluorescence signal as described (*64*). Levels of immunoprecipitated PTPN22 were evaluated by Western blotting. For intracellular dephosphorylation assay, 3× FLAG PTPN22 WT, S449A, D195A/C227S (DACS PTPN22 WT), or D195A/C227S/S449A (DACS PTPN22 S449A) PTPN22 were cotransfected with hemagglutinin-tagged ZAP70 (HA-ZAP70) or untagged LCK into HEK293T or COS-7 cells. The cells were washed twice with cold phosphate-buffered saline (PBS) and harvested for Western blot analysis at 48 hours.

### Samples preparation for phospho-MS

KO or WT Jurkat cell lines were starved for 48 hours and then stimulated for different purposes. For mapping PTPN22 phosphorylation sites, KO or WT Jurkat cells were stimulated with antibodies (1 μg/ml) against human CD3 and CD28 for 5 min and harvested, and PTPN22 protein was affinity-purified using anti-FLAG M2 magnetic beads (Sigma-Aldrich). The immunoprecipitated (IPed) proteins were dissociated from beads by incubation with FLAG peptide (1 mg/ml) in 50 mM tris base (pH 7.4) and 150 mM NaCl buffer at 4°C for 1 hour with rotation. The eluted proteins were collected and confirmed by protein gel electrophoresis, Coomassie blue staining, and Western blot.

### MS-based data acquisition

Samples were loaded into a Thermo Scientific Easy-nLC 1000 coupled to an Acclaim PepMap c18 column and an EasySpray NG (Thermo Fisher Scientific). Mass spectrometer acquisition methods are detailed in Lapek *et al.* (*65*). Briefly, samples loaded onto column were subjected to increasing % organic solvent over the course of 180-min gradient method. Data were acquired using a Thermo Fisher Scientific Orbitrap Fusion and uploaded to ProteomeXchange.

### IP, pull-down, and Western blot

Immunoprecipitation (IP) and Western blot were performed as described (*24*). Briefly, the cells were lysed in 1× TNE lysis buffer [50 mM tris base (pH 7.4), 150 mM NaCl, and 1 mM EDTA] supplied with proteinase inhibitors and 1 mM phenylmethylsulfonyl fluoride. After removing the cell debris by centrifuging at 15,000 rpm for 10 min at 4°C, the supernatants were collected and subjected to IP or pull-down using the indicated antibodies. The IPed proteins were separated by SDS-PAGE and transferred to nitrocellulose membranes. Target proteins were probed with specific antibodies and visualized by chemiluminescence detection system.

### Dual-luciferase reporter assay

The dual-luciferase reporter assay was performed as previously described (*24*).

### Proximity ligation assay

The Duolink PLA was performed by using Duolink In Situ Red Starter Kit mouse/rabbit according to Duolink In Situ Fluorescence protocol (Sigma-Aldrich). Briefly, coverslips were precoated with anti-human CD3 antibody (1 μg/ml) or ICAM-1 (3 μg/ml) at 4°C overnight. Jurkat cells (3× 10^6^) were added onto the coverslips and stimulated at 37°C for indicated time. For TCR stimulation–induced PLA, Jurkat cells were stimulated together with soluble anti-human CD28 antibody (1 μg/ml) for 30 min. For ICAM-1–induced PLA, the cells were stimulated for 20 min. The cells were then fixed with 4% paraformaldehyde in PBS for 15 min at room temperature, followed by permeabilization with 0.2% Triton X-100 for 10 min. After three washes in cold PBS, PLA assay was performed following the standard protocol using anti-FLAG M2 (mouse), anti-ZAP70 (rabbit), anti-LCK (rabbit or mouse), or anti-CD45 (rabbit) antibodies. Images of PLA signal (red dots) were acquired using an Echo Revolve fluorescence microscope, and quantification was performed using ImageJ.

### siRNA knockdown assay

siRNA knockdown assay was performed as previously described (*24*). In brief, ON-TARGETplus SMARTpool siRNA-targeting human PRKACA, CD45, ZAP70, CSK, or ON-TARGETplus Nontargeting Pool siRNA (Dharmacon) at a concentration of 50 nM were nucleofected into 20 × 10^6^ Jurkat cells in six-well plates using the Human T Cell Nucleofector Kit (Lonza) according to the manufacturer’s instructions using program T-23 on an Amaxa Nucleofector II. Transfected cells were harvested for Western blot analysis after 48 hours, and expression of target proteins was calculated with ImageJ.

### Flow cytometry and cell sorting

Flow cytometry and cell sorting were performed following described protocols (*66*). For surface markers staining, single-cell suspensions were prepared and washed twice with fluorescence-activated cell sorting buffer (2.5% FBS and 1 mM EDTA), blocked with human or mouse Fc blocker (BD Biosciences) for 15 min at room temperature and stained with fluorochrome-conjugated antibodies: CD3 (17A2), CD19 (6D5), CD44 (IM7), and CD69 (FN50, H1.2F3) antibodies were purchased from BioLegend. PD-1 (RMP1-30), F4/80 (BM8), GL7 (GL-7), CD25 (PC61.5), CD62L (MEL-14), TCR-β (H57-597), CD8 (53-6.7), and CD4 (RM4-5) antibodies were purchased from Thermo Fisher Scientific. Intracellular staining was performed with the intracellular fixation buffer, permeabilization buffer, and the Foxp3/Transcription Factor Staining Buffer Set (Thermo Fisher Scientific). For intracellular staining of transcription factors, antibodies recognizing FoxP3 (FJK-16 s) and Bcl6 (BCL-DWN) were obtained from Thermo Fisher Scientific. Dead cells were excluded from analysis by staining with Fixable Viability dye from Thermo Fisher Scientific. Cells were assessed by a ZE5 (Bio-Rad) or ID7000 (SONY) flow cytometer. Results were analyzed by FlowJo software (Treestar, Ashland, OR). Targeted cell populations were sorted by a SONY SH800 or a BD Symphony S6 Cell Sorter. For thymic immunophenotyping, thymocytes from either 4-week-old *Ptpn22*^*WT*^ or *Ptpn22*^*S452A*^ mice were collected, and single-cell suspensions were used to analyze the percentage of CD4^−^CD8^−^ double-negative, CD4^+^ single-positive, CD8^+^ single-positive, and CD4^+^CD8^+^ double-positive populations.

For CD4^+^ T cell proliferation, CD4^+^ T cells were isolated from spleens of either 8-week-old *Ptpn22*^*WT*^ or *Ptpn22*^*S452A*^ female mice using the EasySep Mouse CD4^+^ T cell isolation kit (STEMCELL Technologies) as previously described (*66*). Isolated CD4^+^ T cells were stimulated with Dynabeads mouse T-Activator CD3/CD28 in the presence of Il-2 (10 ng/ml) for 3 days. Next, the total cell numbers were counted, and T cell proliferation was assessed by staining equal amounts of T cells with CellTrace Violet dye (Thermo Fisher Scientific) for 3 to 4 days together with mouse CD3/CD28 Dynabeads and Il-2 (10 ng/ml). After removal of Dynabeads, the cells were harvested, washed, stained with Fixable Viability Dye 780 (Thermo Fisher Scientific), and analyzed by flow cytometry.

For the CD69 activation assay, mouse CD4^+^ T cells were isolated and stimulated as described above. The cells (1 × 10^6^) were cultured in plates uncoated or coated with anti-CD3 (0.5 μg/ml) antibodies in complete RPMI 1640 media containing soluble anti-CD28 (1 μg/ml) antibodies, (R&D Systems) for 4 hours. The cells were then collected, stained with fluorescein isothiocyanate anti-mouse CD69 antibody and Fixable Viability Dye 780 and analyzed by flow cytometry.

### Optimized scRNA sequencing

For optimized scRNA sequencing, J.Lck WT or J.Lck Y192F cells were electroporated with plasmids encoding mEGFP-tagged and C-terminal amino acid codon sequence optimized WT or S449A PTPN22, respectively, for 24 hours. The cells were stimulated with anti-human CD3/CD28 antibodies (0.5 μg/ml) for 12 hours. mEGFP^+^ cells were sorted with a BD Symphony S6 Cell Sorter and subjected to 3′ single-cell and CITE-seq by using TotalSeq-B0146 anti-human CD69 Antibody (BioLegend). Next, mEGFP^+^ cells were subjected to library preparation and sequencing under illumina Novaseq X plus system (UCLA). Libraries were constructed using the Chromium Next GEM Single Cell 3′ Kit v3.1 with the 3′ Feature Barcode Kit (10x Genomics) by loading sorted cells to target 11,000 ~ 15,000 cells. 3′ GEX and Feature Barcode libraries were sequenced on the Illumina NovaSeq X Plus platform (2× 100 bp) at a 4:1 ratio.

We quantified mRNA and antibody-derived tag unique molecular identifier (UMI) counts using Cell Ranger v6.1.2 (*67*). Reads were aligned to a custom GRCh38 human reference genome with the addition of the optimized PTPN22 WT and S449A sequences (table S3). The Seurat package was used for quality control and downstream processing of single-cell CITE-seq data. Cells with less than 500 genes or more than 5000 genes expressed were removed. In addition, cells with more than 30,000 UMI were removed along with those that had a mitochondrial content higher than 7%. Further, doublets were removed using DoubletFinder version 2.0.3 (*68*). After QC filtering, a total of 17,677 cells remained across all five conditions. Data were merged, log normalized, and scaled using the ScaleData function with the linear model before dimension reduction with RunPCA. Clusters were identified with the FindNeighbors function constructing a KNN graph based on the Euclidean distance in principal components analysis space (based on the first 14 principal components) with edge weight between cells based on their Jaccard similarity. Following this, modularity optimization with the Louvain algorithm, to iteratively group cells together, was performed using the FindClusters function with a resolution of 0.1. The Uniform Manifold Approximation and Projection embeddings were then calculated and plotted. Differential expression analysis to compare between genotypes was conducted using the Findmarkers function.

### Phospho-ERK, phospho-SLP-76, and CD69 activation assay

The J.OT1.hCD8^+^ or its derivatives with PTPN22 S449A variant (J.OT1 N22 WT or J.OT1 N22 449) were incubated with peptide-pulsed T2-K^b^ cells as previously described with minor modifications (*44*). Briefly, 1 × 10^6^ T2-K^b^ cells were pulsed with a series of titrated concentrations of OVA or altered peptide ligand (APL) peptides and incubated at 37°C for 1 hour in flat-bottom 96-well plates. The J.OT1 N22 WT or J.OT1 N22 449 cells were added into each well of the 96-well plate that contained the peptide-pulsed T2-K^b^ cells. The plates were incubated at 37°C for the indicated times. OT-II^+^ CD4^+^ T cells isolated from mice expressing *Ptpn22*^WT^ or *Ptpn22*^S452A^ were cultured with 1 × 10^6^ irradiated (35 Gy) splenocytes from RAG2 KO mice as antigen-presenting cells and pulsed with titrated concentrations of OVA or APL peptides at 37°C for 16 hours. The phosphorylation of ERK (Thr^202^/Tyr^204^), SLP-76 (Tyr^128^), and CD69 activation were analyzed by flow cytometry.

### OVA immunization and tissue processing

Complete Freund’s Adjuvant (CFA) + OVA (EK-0301, lot: 125) and Incomplete Freund’s Adjuvant (IFA) + OVA (EK-0311, lot: 125) were purchased from Hooke Laboratories. On day 0, two distinct 50-μl subcutaneous injections of OVA/CFA emulsion were administered in the back of 8-week-old *Ptpn22^WT^* or *Ptpn22^S452A^* mice. On day 10, blood samples were collected by retro-orbital bleeding, and the mice received a subcutaneous booster immunization of 100 μl of OVA/IFA emulsion in the back. On day 21, the mice were bled retro-orbitally and euthanized to isolate the serum for specific IgG testing and to collect axillary lymph nodes for flow cytometry analysis.

### Resiquimod (R848) lupus model and tissue processing

The resiquimod lupus model was performed following a previously published protocol (*46*). Briefly, 8 to 12 weeks old *Ptpn22^WT^* or *Ptpn22^S452A^* littermate mice were treated with resiquimod for four weeks. Proteinuria was monitored by using Albustix urinalysis reagent test strips (Siemens Healthineers) every week. After mouse euthanasia, the spleens, kidneys, and peripheral blood were collected and processed for further experiments. Spleen and body weights were measured to calculate the spleen/body weight ratio. Splenocytes were isolated and counted for flow cytometry analysis. Mouse kidneys were preserved in 10% buffered formalin and embedded into paraffin after tissue processing. The sections were stained with periodic acid–Schiff and imaged using an Echo Revolve microscope. IgG deposition in the kidney was assessed by using Alexa Fluor 647–conjugated anti-mouse IgG antibody (Thermo Fisher Scientific, A-31571). Fluorescent images were taken using the fluorescence microscope (Echo Revolve), and intensity was quantified by ImageJ in a blinded fashion.

### Statistical analysis

Statistical analyses were performed using GraphPad Prism 9.2.0 software by applying one-way and two-way analysis of variance (ANOVA), unpaired *t* test, Kruskal-Wallis test, Kolmogorov-Smirnov test, and Mann-Whitney test as indicated in the figure legends. Error bars represent means ± SEM from at least three biological repeats. Significant differences were defined as *P < 0.05, **P < 0.01, ***P < 0.001.

## Supplementary Materials

### This PDF file includes:

Figs. S1 to S8 Tables S1 to S4 Uncropped blots in Figs. 1K, 3C, 4G, and 5C
