# Supplementary material for: PTPN22-CD45 dual phosphatase retrograde feedback enhances TCR signaling and autoimmunity
Source: Sci Adv. 2025 Sep 5;11(36):eadw2568. doi: 10.1126/sciadv.adw2568 (PMC12412669; doi:10.1126/sciadv.adw2568)

Science Advances
AAAS

Supplementary Materials for

PTPN22-CD45 dual phosphatase retrograde feedback enhances TCR signaling and autoimmunity

Shen Yang *et al.*

Corresponding author: Nunzio Bottini, nunzio.bottini@cshs.org

Sci. Adv. **11**, eadw2568 (2025)
DOI: 10.1126/sciadv.adw2568

This PDF file includes:

Figs. S1 to S8
Tables S1 to S4
Uncropped blots in Figs. 1K, 3C, 4G, and 5C

fig. S1

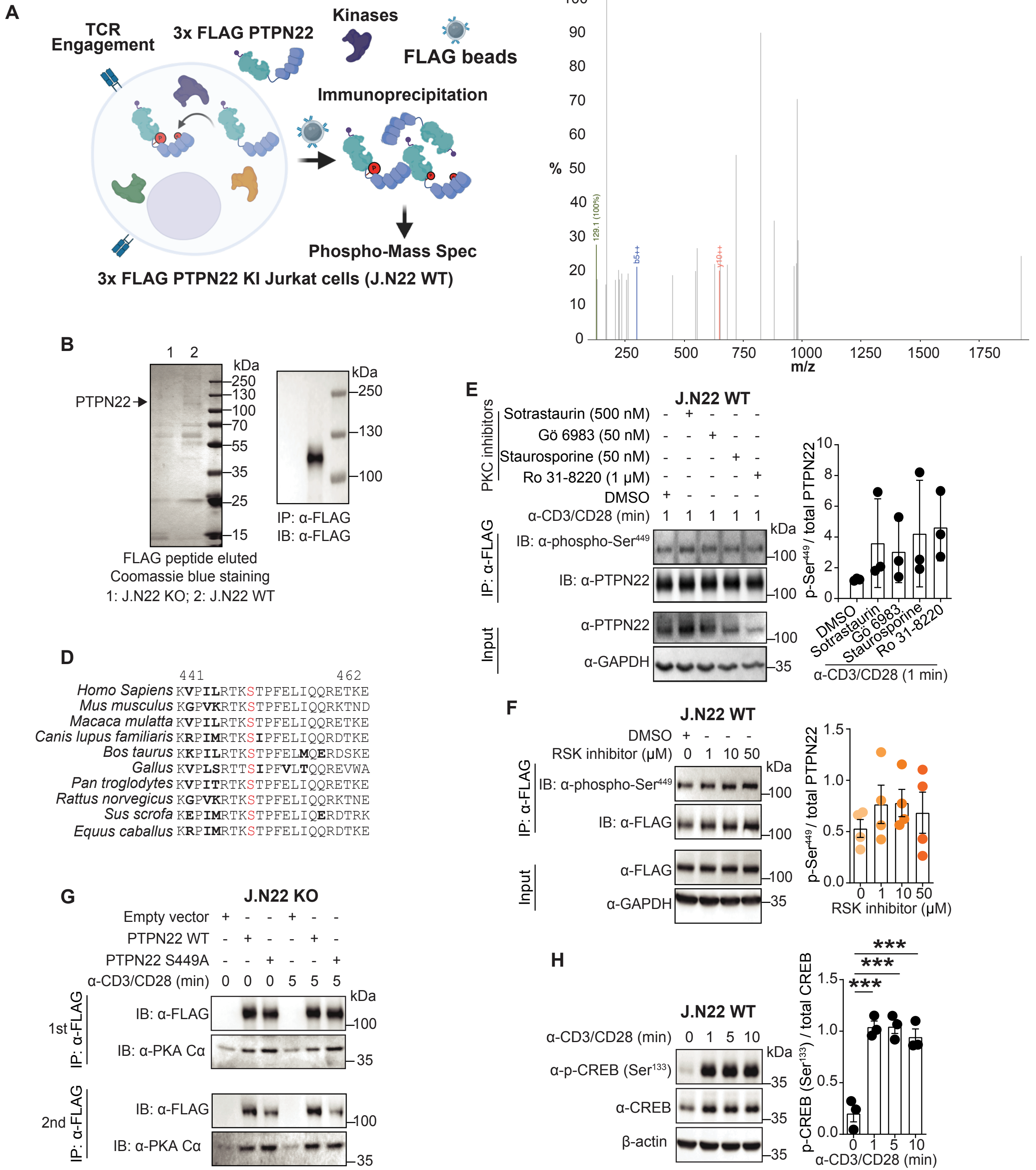
A
TCR Engagement
3x FLAG PTPN22
Kinases
FLAG beads
Immunoprecipitation
Phospho-Mass Spec
3x FLAG PTPN22 KI Jurkat cells (J.N22 WT)
B
PTPN22
kDa
FLAG peptide eluted
Coomassie blue staining
1: J.N22 KO; 2: J.N22 WT
IP: α-FLAG
IB: α-FLAG
C
TKSTPFELIQQR,MH+ 1527.7567
Spectrum m/z 654.0002, Charge 3, MH+ 195.9849
%
m/z
D
441 462
Homo Sapiens KVPILRTKSTPFELIQQRETKE
Mus musculus KGPVKRTKSTPFELIQQRKTND
Macaca mulatta KVPILRTKSTPFELIQQRETKE
Canis lupus familiaris KRPIMRTKSIPFELIQQRDTKE
Bos taurus KKPILRTKSTPFELMQERDSKE
Gallus KVPLSRTTSIPFVLTQQREVWA
Pan troglodytes KVPITRTKSTPFELIQQRETKE
Rattus norvegicus KGPVKRTKSTPFELIQQRKTNE
Sus scrofa KEPIMRTKSTPFELIQERDTRK
Equus caballus KRPIMRTKSTPFELIQQRDTKE
E
J.N22 WT
PKC inhibitors
Sotrastaurin (500 nM)
Gö 6983 (50 nM)
Staurosporine (50 nM)
Ro 31-8220 (1 μM)
DMSO
α-CD3/CD28 (min)
IP: α-FLAG
IB: α-phospho-Ser449
IB: α-PTPN22
Input
α-PTPN22
α-GAPDH
p-Ser449 / total PTPN22
DMSO
Sotrastaurin
Gö 6983
Staurosporine
Ro 31-8220
α-CD3/CD28 (1 min)
F
J.N22 WT
DMSO
RSK inhibitor (μM)
IP: α-FLAG
IB: α-phospho-Ser449
IB: α-FLAG
Input
α-FLAG
α-GAPDH
p-Ser449 / total PTPN22
RSK inhibitor (μM)
G
J.N22 KO
Empty vector
PTPN22 WT
PTPN22 S449A
α-CD3/CD28 (min)
1st IP: α-FLAG
IB: α-FLAG
IB: α-PKA Cα
2nd IP: α-FLAG
IB: α-FLAG
IB: α-PKA Cα
H
J.N22 WT
α-CD3/CD28 (min)
α-p-CREB (Ser133)
α-CREB
β-actin
p-CREB (Ser133) / total CREB
***
***
***
α-CD3/CD28 (min)

fig. S1. Validation of kinases responsible for PTPN22 Ser^{449} phosphorylation

(**A**) Schematic illustration of 3× FLAG PTPN22 protein purification from J.N22 KO or WT Jurkat cells after TCR engagement. (**B**) FLAG-tagged PTPN22 immunoprecipitated with anti-FLAG M2 beads and eluted with FLAG peptides stained with Coomassie blue (left) or an anti-FLAG antibody in a Western blot (right). (**C**) Mass spectrometry graph of phosphopeptide including residue Ser^{449} identified by analysis of PTPN22 purified from TCR stimulated J.N22 WT cells. The phosphorylation site is labeled in red. (**D**) Alignment of residues surrounding PTPN22 Ser^{449} (red) from various species, numbered according to human PTPN22. (**E**) PTPN22 Ser^{449} phosphorylation in J.N22 WT cells treated with DMSO or PKC inhibitors (Sotrastaurin, Gö 6983, Staurosporine, Ro 31-8220), unstimulated or stimulated with anti-CD3/CD28 antibodies for 1 min, left panel is representative of three independent experiments. (**F**) PTPN22 Ser^{449} phosphorylation in J.N22 WT cells treated with DMSO or RSK inhibitor (LJH685, 0, 1, 10, 50 mM) for 30 min, evaluated by Western blotting, left panel is representative of four independent experiments. (**G**) Co-immunoprecipitation analysis of the interaction between PTPN22 and PKA Cα in J.N22 KO cells electroporated with Empty vector or a plasmid encoding 3× FLAG tagged PTPN22 WT or S449A and stimulated with or without antibodies against human CD3/CD28. Representative of two independent experiments (**H**) CREB Ser^{133} phosphorylation in J.N22 WT cells treated with anti-CD3/CD28 antibodies for 0, 1, 5, 10 min. Left panel is representative of three independent experiments. Data are presented as mean ± SEM. Phospho-protein histograms show quantification of phospho-protein/total protein ratio. Statistical significance was assessed by one-way ANOVA followed by Dunnett's post hoc test (**E**,**F**,**H**). ***P<0.001.

fig. S2

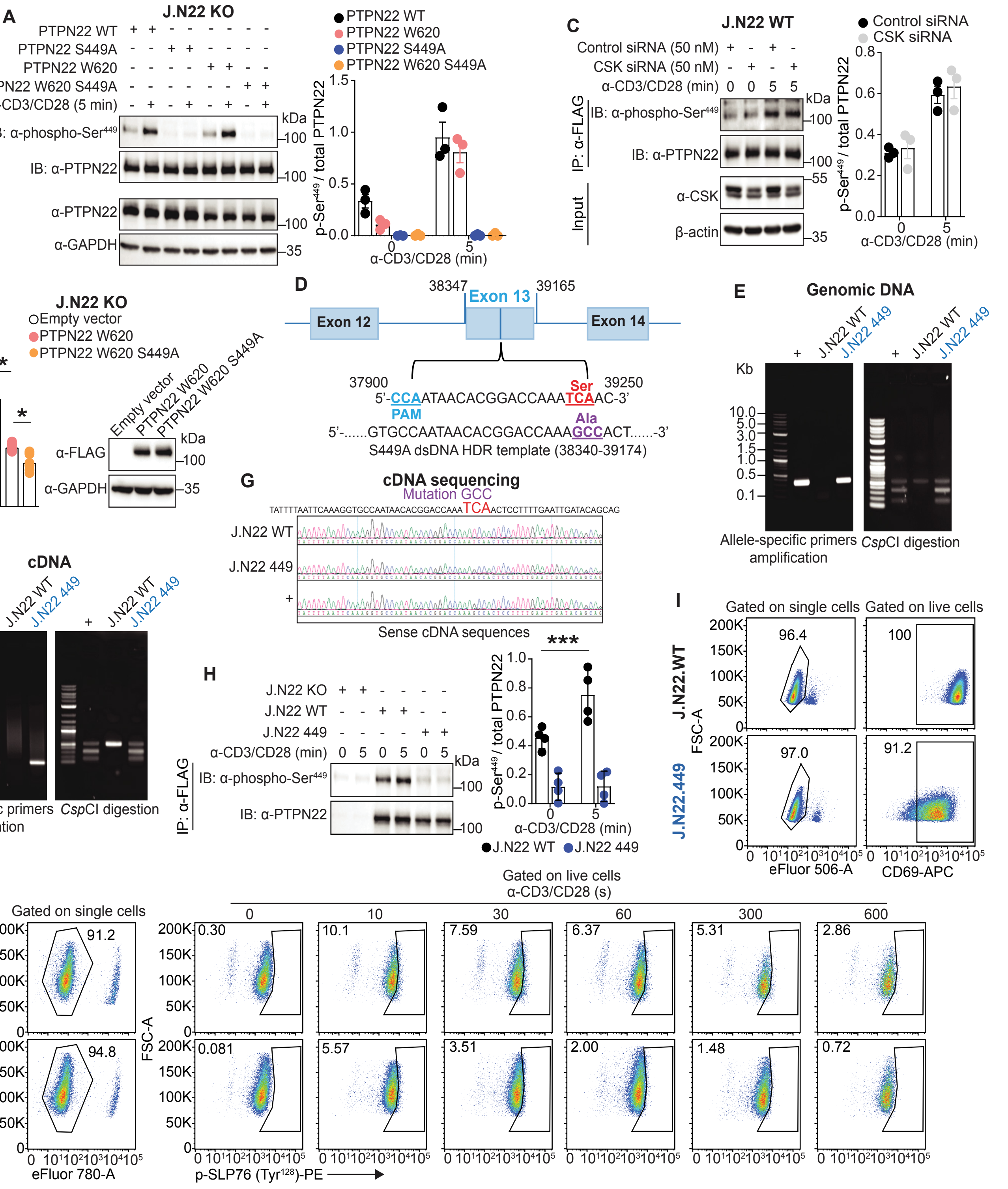
A
J.N22 KO
PTPN22 WT
PTPN22 S449A
PTPN22 W620
PTPN22 W620 S449A
α-CD3/CD28 (5 min)
IP: α-FLAG
IB: α-phospho-Ser449
IB: α-PTPN22
Input
α-PTPN22
α-GAPDH
kDa
p-Ser449 / total PTPN22
α-CD3/CD28 (min)
B
J.N22 KO
Empty vector
PTPN22 W620
PTPN22 W620 S449A
NFAT/AP-1 Luciferase activity
α-FLAG
α-GAPDH
C
J.N22 WT
Control siRNA (50 nM)
CSK siRNA (50 nM)
α-CD3/CD28 (min)
IB: α-phospho-Ser449
IB: α-PTPN22
α-CSK
β-actin
Control siRNA
CSK siRNA
D
Exon 12
Exon 13
Exon 14
38347
39165
37900
39250
5'-CCAATAACACGGACCAAATCAAC-3'
PAM
Ser
Ala
5'-......GTGCCAATAACACGGACCAAAGCCACT......-3'
S449A dsDNA HDR template (38340-39174)
E
Genomic DNA
J.N22 WT
J.N22 449
Kb
Allele-specific primers amplification
CspCI digestion
F
cDNA
G
cDNA sequencing
Mutation GCC
TATTTTAATTCAAAGGTGCCAATAACACGGACCAAATCAACTCCTTTTGAATTGATACAGCAG
J.N22 WT
J.N22 449
Sense cDNA sequences
H
J.N22 KO
J.N22 WT
J.N22 449
α-CD3/CD28 (min)
IP: α-FLAG
IB: α-phospho-Ser449
IB: α-PTPN22
***
I
Gated on single cells
Gated on live cells
J.N22.WT
J.N22.449
FSC-A
eFluor 506-A
CD69-APC
96.4
100
97.0
91.2
J
Gated on single cells
Gated on live cells
α-CD3/CD28 (s)
0
10
30
60
300
600
91.2
94.8
0.30
10.1
7.59
6.37
5.31
2.86
0.081
5.57
3.51
2.00
1.48
0.72
eFluor 780-A
p-SLP76 (Tyr128)-PE

fig. S2. CRISPR/Cas9 mediated S449A KI Jurkat cells construction.

(**A**) Evaluation of PTPN22 Ser^{449} phosphorylation by immunoprecipitation from lysates of J.N22 KO cells electroporated with plasmids encoding FLAG-tagged WT, S449A, W620, or W620 S449A PTPN22 and treated with antibodies against CD3/CD28 for 5 min. (**B**) Evaluation of PTPN22 W620 variant inhibition of TCR signaling by dual-luciferase reporter assay in J.N22 KO cells expressing W620 or W620 S449A PTPN22 and stimulated with antibodies against CD3/CD28. Luciferase activity was measured (left) and normalized to the amount of PTPN22 relative to that of GAPDH as assessed by Western blotting (right). Histogram shows experimental replicates. (**C**) TCR-induced PTPN22 Ser^{449} phosphorylation in CSK knockdown J.N22 WT cells. Histograms show experimental replicates of quantification of the Ser^{449} phosphorylated/total PTPN22 ratio (right). (**D**) Schematic of Cas9 guide RNA (gRNA) designed for replacement of serine 449 on Exon 13 of the PTPN22 genomic DNA locus. (**E** and **F**) Screening of CRISPR/Cas9 mediated S449A KI cell clone by Allele-Specific primer amplification (left) and *Csp*CI digestion (right) using genomic DNA (**E**) or cDNA (**F**) as template. 3× FLAG tagged PTPN22 S449A eukaryotic expression plasmid served as "+" control. (**G**) cDNA sequencing trace of S449A KI positive candidate. cDNA was synthesized using RNA from the candidate cell clones and standard PCR was performed to amplify the target cDNA sequences surrounding Ser^{449}. PTPN22 WT cells and 3× FLAG tagged PTPN22 S449A eukaryotic expression plasmid served as negative or positive (+) controls, respectively. (**H**) Immunoprecipitation analysis of endogenous phospho-PTPN22 Ser^{449} in J.N22 WT or J.N22 449 candidate Jurkat T cells stimulated with or without antibodies against human CD3/CD28 for 5 min. Histograms show experimental replicates of quantification of the Ser^{449} phosphorylated/total PTPN22 ratio (right). (**I** and **J**) Representative gating strategies to detect CD69 expression (**I**) or phosphorylation of SLP76 Tyr^{128} (**J**) in J.N22 WT and J.N22 449 cells stimulated by anti-human CD3/CD28 antibodies. Data are presented as mean ± SEM, and are from three (**A**-**C**) or four (**H**) independent experiments. Representative blots are shown (left). Statistical significance was assessed by two-way ANOVA followed by Bonferroni's post hoc test (**A**,**C**,**H**) and one-way ANOVA followed by Dunnett's post hoc test (**B**). $*P<0.05$, $***P<0.001$.

fig. S3

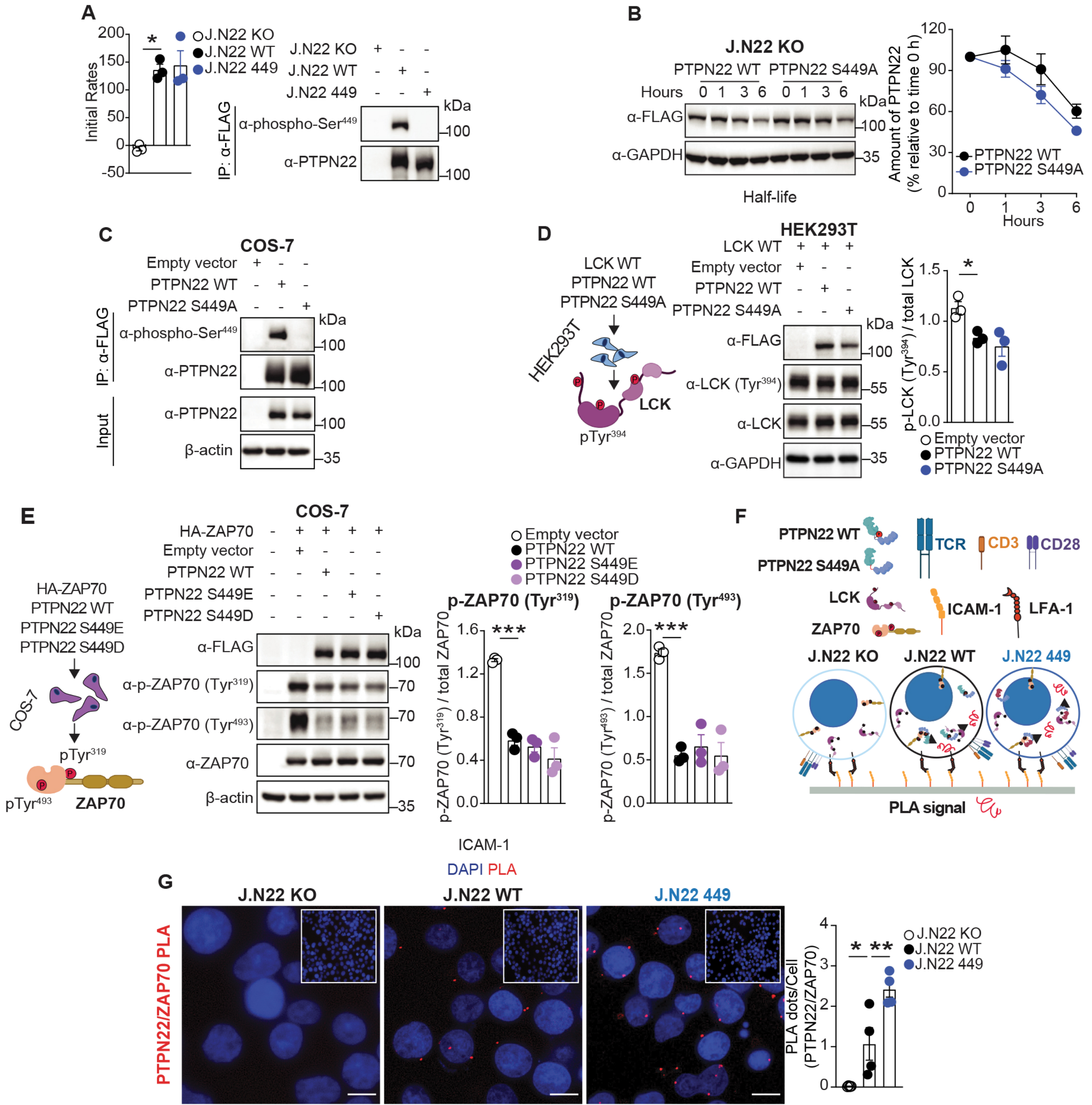

A
Initial Rates
J.N22 KO
J.N22 WT
J.N22 449
IP: α-FLAG
α-phospho-Ser449
α-PTPN22
kDa
B
J.N22 KO
PTPN22 WT
PTPN22 S449A
Hours
α-FLAG
α-GAPDH
Half-life
Amount of PTPN22 (% relative to time 0 h)
Hours
C
COS-7
Empty vector
PTPN22 WT
PTPN22 S449A
IP: α-FLAG
Input
α-phospho-Ser449
α-PTPN22
β-actin
D
HEK293T
LCK WT
Empty vector
PTPN22 WT
PTPN22 S449A
α-FLAG
α-LCK (Tyr394)
α-LCK
α-GAPDH
pTyr394
LCK
p-LCK (Tyr394) / total LCK
E
COS-7
HA-ZAP70
Empty vector
PTPN22 WT
PTPN22 S449E
PTPN22 S449D
α-FLAG
α-p-ZAP70 (Tyr319)
α-p-ZAP70 (Tyr493)
α-ZAP70
β-actin
pTyr319
pTyr493
ZAP70
p-ZAP70 (Tyr319)
p-ZAP70 (Tyr493)
p-ZAP70 (Tyr319) / total ZAP70
p-ZAP70 (Tyr493) / total ZAP70
F
PTPN22 WT
PTPN22 S449A
TCR
CD3
CD28
LCK
ICAM-1
LFA-1
ZAP70
J.N22 KO
J.N22 WT
J.N22 449
PLA signal
ICAM-1
DAPI PLA
G
J.N22 KO
J.N22 WT
J.N22 449
PTPN22/ZAP70 PLA
PLA dots/Cell (PTPN22/ZAP70)

fig. S3. Evaluation of PTPN22^{WT} vs PTPN22^{S449A} phosphatase activity.

(**A**) Phosphatase activity of WT and S449A PTPN22. Assays were performed by using immunoprecipitated PTPN22 from J.N22 WT or J.N22 449 cells and DiFMUP as a substrate. Immunoprecipitated proteins from J.N22 KO cells served as negative control. Histogram shows quantification of initial rates of reaction normalized to the amount of PTPN22 WT as assessed by Western blotting (representative blot in right panel). (**B**) Western blot analysis of PTPN22 half-life in J.N22 KO cells overexpressing FLAG-tagged WT or S449A PTPN22 and treated with CHX for the indicated times (left). The graph (right) shows quantification of PTPN22 normalized to time 0 (=100) after normalizing to GAPDH. (**C**) PTPN22 Ser^{449} phosphorylation in COS-7 cells expressing empty vector, 3× FLAG tagged WT or S449A PTPN22. (**D**) Immunoblotting analysis of HEK293T cells co-transfected with untagged LCK and 3× FLAG tagged WT or S449A PTPN22 (left). Histograms show quantification of the phospho-LCK Tyr^{394} /total LCK ratio (right). (**E**) Dephosphorylation of ZAP70 in COS-7 cells co-transfected with HA tagged ZAP70 and 3× FLAG tagged WT, S449E or S449D PTPN22 assessed by immunoblotting (left). Histograms show quantification of the phospho-ZAP70 Tyr^{319} or Tyr^{493} normalized to total ZAP70 ratio. (**F** and **G**) PLA was performed between PTPN22 and ZAP70 in J.N22 WT and J.N22 449 cells using mouse anti-FLAG and rabbit anti-ZAP70 antibodies after ICAM-1 stimulation (**G**). Images of PLA (red, left) and quantification of PLA are shown (right). Scale bars, 10 µm. Data are presented as mean ± SEM and are from three (**A**-**E**) or four (**G**) independent experiments. Statistical significance was assessed by one-way ANOVA followed by Dunnett's post hoc test (**A**,**D**,**E**,**G**) and two-factor repeated-measures ANOVA (**B**). *P <0.05, **P <0.01, ***P<0.001.

fig. S4

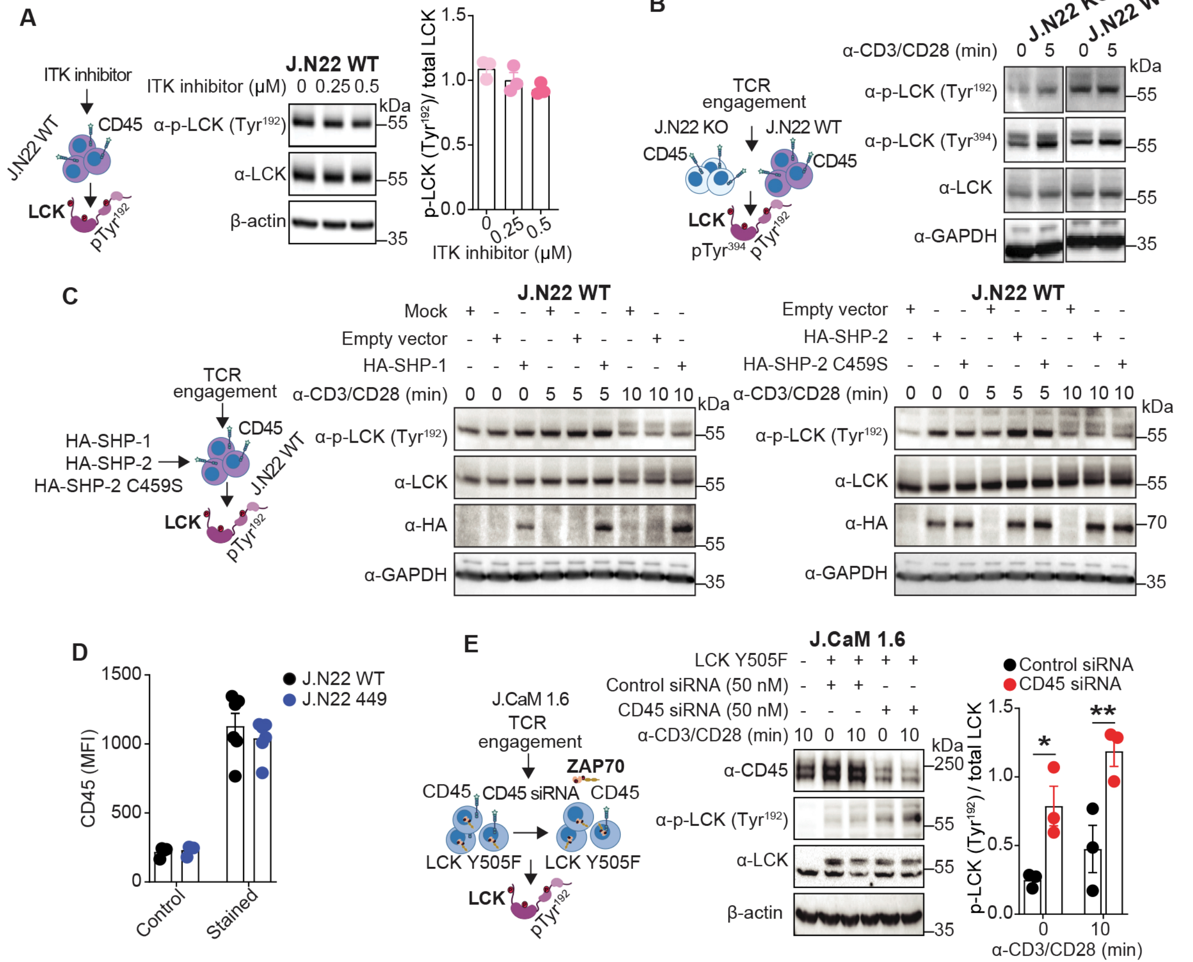
A
ITK inhibitor
J.N22 WT
CD45
LCK
pTyr192
J.N22 WT
ITK inhibitor (μM) 0 0.25 0.5
α-p-LCK (Tyr192)
α-LCK
β-actin
kDa
55
55
35
p-LCK (Tyr192)/ total LCK
ITK inhibitor (μM)
B
J.N22 KO
J.N22 WT
α-CD3/CD28 (min) 0 5 0 5
TCR engagement
CD45
LCK
pTyr394
pTyr192
α-p-LCK (Tyr192)
α-p-LCK (Tyr394)
α-LCK
α-GAPDH
C
J.N22 WT
Mock
Empty vector
HA-SHP-1
α-CD3/CD28 (min) 0 0 0 5 5 5 10 10 10
TCR engagement
CD45
HA-SHP-1
HA-SHP-2
HA-SHP-2 C459S
J.N22 WT
LCK
pTyr192
α-p-LCK (Tyr192)
α-LCK
α-HA
α-GAPDH
J.N22 WT
Empty vector
HA-SHP-2
HA-SHP-2 C459S
α-CD3/CD28 (min) 0 0 0 5 5 5 10 10 10
kDa
70
D
J.N22 WT
J.N22 449
CD45 (MFI)
Control
Stained
E
J.CaM 1.6
LCK Y505F
Control siRNA (50 nM)
CD45 siRNA (50 nM)
α-CD3/CD28 (min) 10 0 10 0 10
J.CaM 1.6
TCR engagement
ZAP70
CD45
CD45 siRNA
LCK Y505F
LCK
pTyr192
α-CD45
α-p-LCK (Tyr192)
α-LCK
β-actin
250
Control siRNA
CD45 siRNA
p-LCK (Tyr192) / total LCK
*
**
α-CD3/CD28 (min)

fig. S4. Assessment of kinases and phosphatases regulating LCK Tyr^{192} phosphorylation.

(**A**) LCK Tyr^{192} phosphorylation was analyzed in J.N22 WT cells treated with 0, 0.25, and 0.5 μM ITK inhibitor (BMS-509744). (**B**) LCK Tyr^{192} phosphorylation in KO and WT cells without or with TCR stimulation analyzed by Western blotting. (**C**) LCK Tyr^{192} phosphorylation in J.N22 WT cells overexpressing HA-SHP-1, HA-SHP-2 or HA-SHP-2 C459S. Cells were stimulated with antibodies against CD3/CD28 for 0, 5, and 10 min. (**D**) CD45 expression in J.N22 WT and 449 cells was analyzed by flow cytometry and evaluated by MFI of cells. (**E**) LCK Tyr^{192} phosphorylation in CD45 knockdown J.CaM 1.6 cells overexpressing Y505F mutant LCK. Cells were stimulated with antibodies against CD3/CD28 antibodies for 0 or 10 min. Data are presented as mean ± SEM and are from three (**A** and **E**), two (**B** and **C**), and six (**D**) independent experiments. Representative blots are shown. Phospho-protein histograms show quantification of phospho-protein/total protein ratio (right). Statistical significance was assessed by one-way ANOVA followed by Dunnett's post hoc test (**A**) and by two-way ANOVA followed by Bonferroni's post hoc test (**D** and **E**). $^{*}P<0.05$, $^{**}P<0.01$.

fig. S5

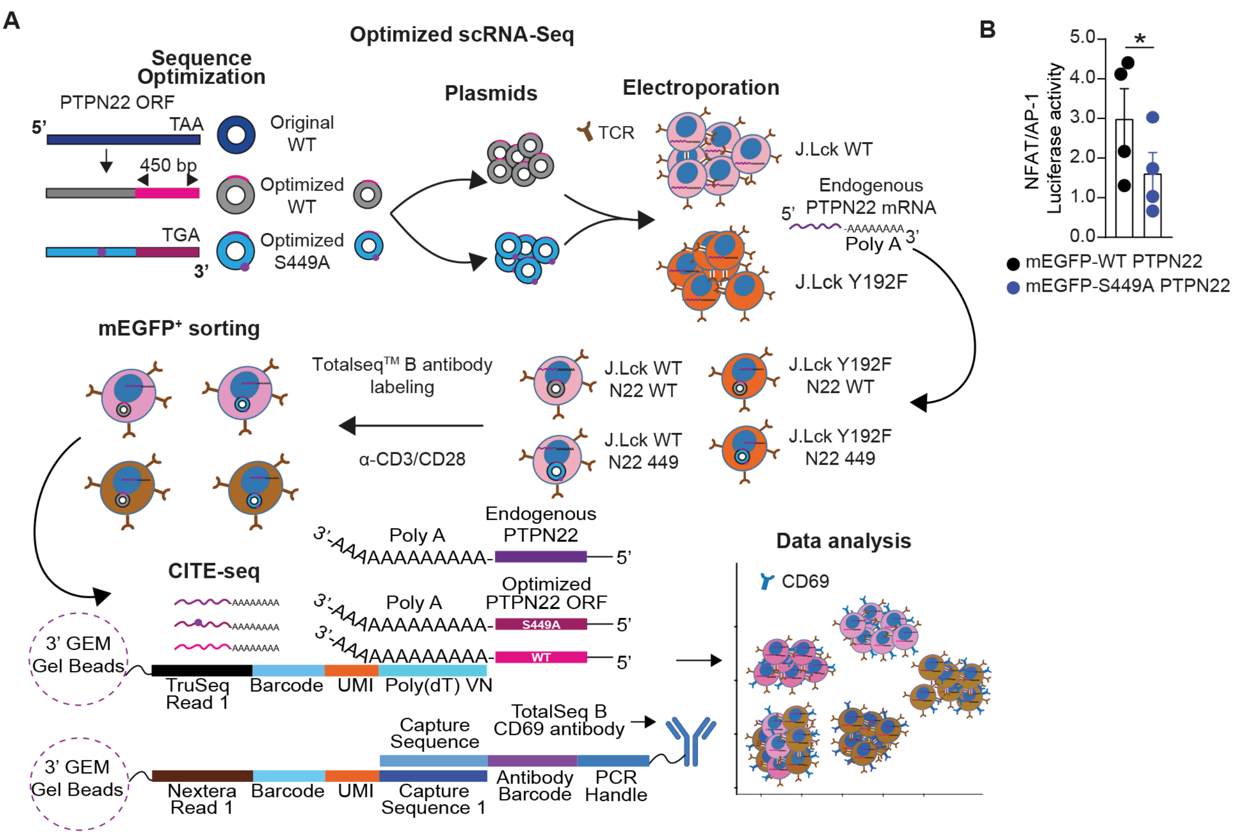
A
Optimized scRNA-Seq
Sequence Optimization
PTPN22 ORF
5'
TAA
450 bp
TGA
3'
Original WT
Optimized WT
Optimized S449A
Plasmids
Electroporation
TCR
J.Lck WT
Endogenous PTPN22 mRNA
5'
AAAAAAAA
3'
Poly A
J.Lck Y192F
mEGFP+ sorting
Totalseq™ B antibody labeling
α-CD3/CD28
J.Lck WT N22 WT
J.Lck Y192F N22 WT
J.Lck WT N22 449
J.Lck Y192F N22 449
Endogenous PTPN22
Poly A
3'-AAAAAAAAAAAAA-
5'
CITE-seq
Optimized PTPN22 ORF
S449A
WT
3' GEM Gel Beads
TruSeq Read 1
Barcode
UMI
Poly(dT) VN
Capture Sequence
TotalSeq B CD69 antibody
Nextera Read 1
Capture Sequence 1
Antibody Barcode
PCR Handle
Data analysis
CD69
B
NFAT/AP-1 Luciferase activity
5.0
4.0
3.0
2.0
1.0
0.0
*
mEGFP-WT PTPN22
mEGFP-S449A PTPN22

fig. S5. Optimized scRNA sequencing in J.Lck cells.

(**A**) Schematic of optimized scRNA-seq and CITE-seq in J.Lck WT or J.Lck Y192F cells expressing either optimized mEGFP PTPN22 WT or S449A mutant. (**B**) Dual-luciferase reporter assay in mEGFP-PTPN22-WT or S449A mutant in J.Lck WT cells. Cells were co-electroporated with PTPN22 variant plasmids and NFAT/AP-1 firefly luciferase and *Renilla* luciferase reporters. Luciferase activity was measured and the firefly/*Renilla* ratio was normalized to the amount of PTPN22 relative to that of GAPDH. Data are presented as mean ± SEM from four independent experiments (**B**). Statistical significance was assessed by two-tailed Mann-Whitney test (**B**). $^*P < 0.05$.

fig. S6

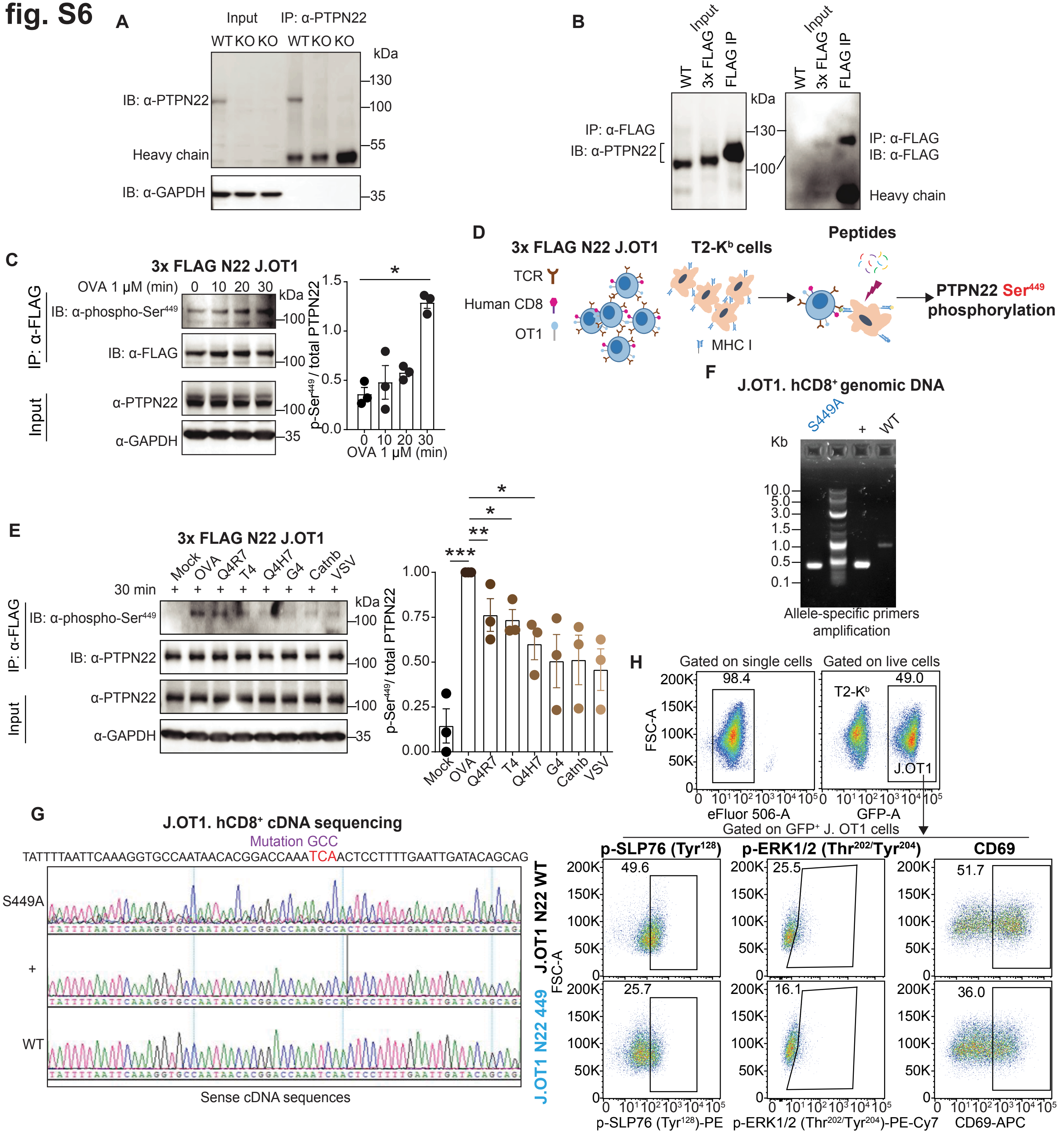

A
Input
IP: α-PTPN22
WT KO KO WT KO KO
kDa
130
100
55
35
IB: α-PTPN22
Heavy chain
IB: α-GAPDH
B
WT
Input
3x FLAG
FLAG IP
kDa
IP: α-FLAG
IB: α-PTPN22
130
100
IP: α-FLAG
IB: α-FLAG
Heavy chain
C
3x FLAG N22 J.OT1
OVA 1 μM (min) 0 10 20 30
IP: α-FLAG
IB: α-phospho-Ser449
IB: α-FLAG
Input
α-PTPN22
α-GAPDH
p-Ser449 / total PTPN22
OVA 1 μM (min)
D
3x FLAG N22 J.OT1
T2-Kb cells
Peptides
TCR
Human CD8
OT1
MHC I
PTPN22 Ser449 phosphorylation
E
3x FLAG N22 J.OT1
Mock OVA Q4R7 T4 Q4H7 G4 Catnb VSV
30 min
IB: α-phospho-Ser449
IB: α-PTPN22
α-PTPN22
α-GAPDH
p-Ser449/ total PTPN22
F
J.OT1. hCD8+ genomic DNA
S449A + WT
Kb
10.0
5.0
3.0
1.5
1.0
0.5
0.1
Allele-specific primers amplification
G
J.OT1. hCD8+ cDNA sequencing
Mutation GCC
TATTTTAATTCAAAGGTGCCAATAACACGGACCAAATCAACTCCTTTTGAATTGATACAGCAG
S449A
+
WT
Sense cDNA sequences
H
Gated on single cells
Gated on live cells
98.4
49.0
T2-Kb
J.OT1
FSC-A
eFluor 506-A
GFP-A
Gated on GFP+ J. OT1 cells
p-SLP76 (Tyr128)
p-ERK1/2 (Thr202/Tyr204)
CD69
J.OT1 N22 WT
J.OT1 N22 449
49.6
25.5
51.7
25.7
16.1
36.0
p-SLP76 (Tyr128)-PE
p-ERK1/2 (Thr202/Tyr204)-PE-Cy7
CD69-APC

fig. S6. J.OT1.hCD8$^+$ cells modification.

(**A** and **B**) Construction of PTPN22 KO (J.OT1 N22 KO), and 3× FLAG tagged KI PTPN22 (3× FLAG N22 J.OT1) J.OT1.hCD8$^+$ Jurkat cells. Immunoprecipitation analysis of PTPN22 expression in J.OT1 N22 KO candidates (**A**). Immunoprecipitation analysis of 3× FLAG tagged PTPN22 expression in 3× FLAG N22 J.OT1candidates (**B**). (**C**) Immunoprecipitation analysis of Ser^{449} phosphorylation in 3× FLAG N22 J.OT1 cells stimulated with 1 μM OVA peptide-pulsed T2-K^b cells for 0, 10, 20, or 30 min. Histogram shows quantification of PTPN22 Ser^{449} phosphorylation normalized to PTPN22 protein (right). (**D**) Schematic illustration of the interaction of 3× FLAG N22 J.OT1 cells with antigen presenting cells pulsed by multiple peptides. (**E**) Ser^{449} phosphorylation in 3× FLAG N22 J.OT1 cells. T2-K^b antigen-presenting cells were pulsed with or without OVA, Q4R7, T4, Q4H7, G4, Catnb, or VSV peptides at a concentration of 1 μM for 1 h. Cells were then stimulated with pulsed T2-K^b cells for 30 min. Ser^{449} phosphorylation on immunoprecipitated PTPN22 was assessed by Western blotting. Histogram shows quantification of phosphorylated Ser^{449} normalized to total PTPN22 protein and OVA peptide stimulated sample (right). (**F**) Allele-specific primer amplification for screening of CRISPR/Cas9 mediated S449A KI PTPN22 J.OT1.hCD8$^+$ cells (J.OT1 N22 449) using genomic DNA as template. (**G**) cDNA sequencing trace for J.OT1. PTPN22 S449A KI positive candidate. cDNA was synthesized using RNA from the candidate cell clones and standard PCR was performed to obtain the target cDNA sequences surrounding Ser^{449}. PTPN22 WT cells and 3× FLAG tagged S449A PTPN22 eukaryotic expression plasmid served as negative and positive control, respectively. (**H**) Representative gating strategy to quantify phosphorylation of p-SLP76 Tyr^{128}, p-ERK1/2 Thr^{202}Tyr^{204}, and expression of CD69 in J.OT1 N22 WT or J.OT1 N22 449 cells stimulated by anti-human CD3/CD28 antibodies. Data in histogram is presented as mean ± SEM. Data are from three independent experiments and representative blots are shown. Statistical significance was assessed by one-way ANOVA followed by Dunnett's post hoc test (**C** and **E**). **P* <0.05, ***P*<0.01, ****P*<0.001.

fig. S7

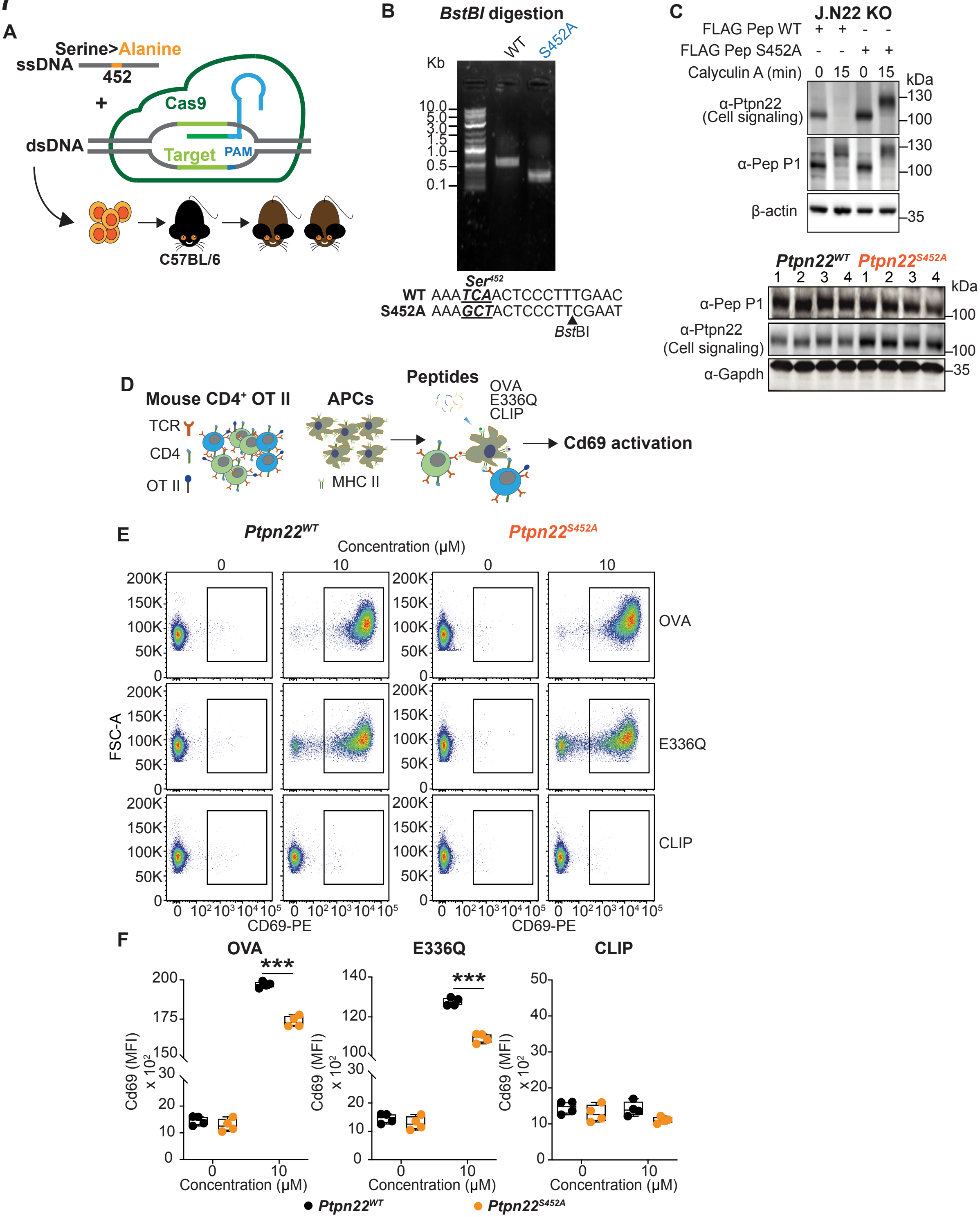
A
Serine>Alanine
ssDNA
452
Cas9
dsDNA
Target
PAM
C57BL/6
B
BstBI digestion
WT
S452A
Kb
10.0
5.0
3.0
1.5
1.0
0.5
0.1
Ser452
WT AAATCAACTCCCTTTGAAC
S452A AAAGCTACTCCCTTCGAAT
BstBI
C
J.N22 KO
FLAG Pep WT
FLAG Pep S452A
Calyculin A (min)
α-Ptpn22 (Cell signaling)
α-Pep P1
β-actin
kDa
Ptpn22WT
Ptpn22S452A
α-Gapdh
D
Mouse CD4+ OT II
APCs
Peptides
OVA
E336Q
CLIP
TCR
CD4
OT II
MHC II
Cd69 activation
E
Concentration (μM)
FSC-A
CD69-PE
F
OVA
E336Q
CLIP
Cd69 (MFI) x 10²
Concentration (μM)
***
Ptpn22WT
Ptpn22S452A

fig. S7. $CD4^+$ T cells from CRISPR/Cas9 mediated *$Ptpn22^{S452A}$* mice exhibit lower responsiveness to antigen stimulation.

(**A**) An illustration of CRISPR/Cas9 mediated generation of the *$Ptpn22^{S452A}$* KI mouse model. (**B**) Screening of CRISPR/Cas9 mediated S452A KI mice by PCR amplification and *Bst*BI digestion using mouse genomic DNA. The WT and S452A KI genomic DNA sequences are shown below. (**C**) Western blot analysis of Ser^{452} phosphorylation in J.N22 KO cells overexpressing 3× FLAG tagged mouse Ptpn22 WT (Pep WT) or S452A (Pep S452A) mutant stimulated with or without calyculin A (upper), and Ptpn22 expression in *$Ptpn22^{WT}$* (n=4) and *$Ptpn22^{S452A}$* (n=4) mice (lower) using anti-Pep P1 antibody (Genentech) and PTPN22 (D6D1H) monoclonal antibody (Cell Signaling Technology). (**D**) An illustration of the interaction of $OT\text{-}II^+$ $CD4^+$ T cells with APCs pulsed by various peptides. (**E** and **F**) Cd69 activation of $OT\text{-}II^+$ $CD4^+$ T cells from 8-week-old *$Ptpn22^{WT}$* (n=4) or *$Ptpn22^{S452A}$* (n=4) mice activated by APCs pulsed with peptides. Splenocytes from RAG2 KO mice were irradiated and used as APCs. $OT\text{-}II^+$ $CD4^+$ T cells were stimulated with OVA, E336Q, or CLIP peptides-pulsed splenocytes at concentrations of 0 μM and 10 μM for 16 h. The activation of Cd69 was analyzed by flow cytometry (**E**) and evaluated by MFI of cells from each mouse (**F**). Data are presented as mean ± SEM and statistical significance was assessed by two-way ANOVA followed by Bonferroni's post hoc test (**F**). $^{***}P<0.001$.

fig. S8

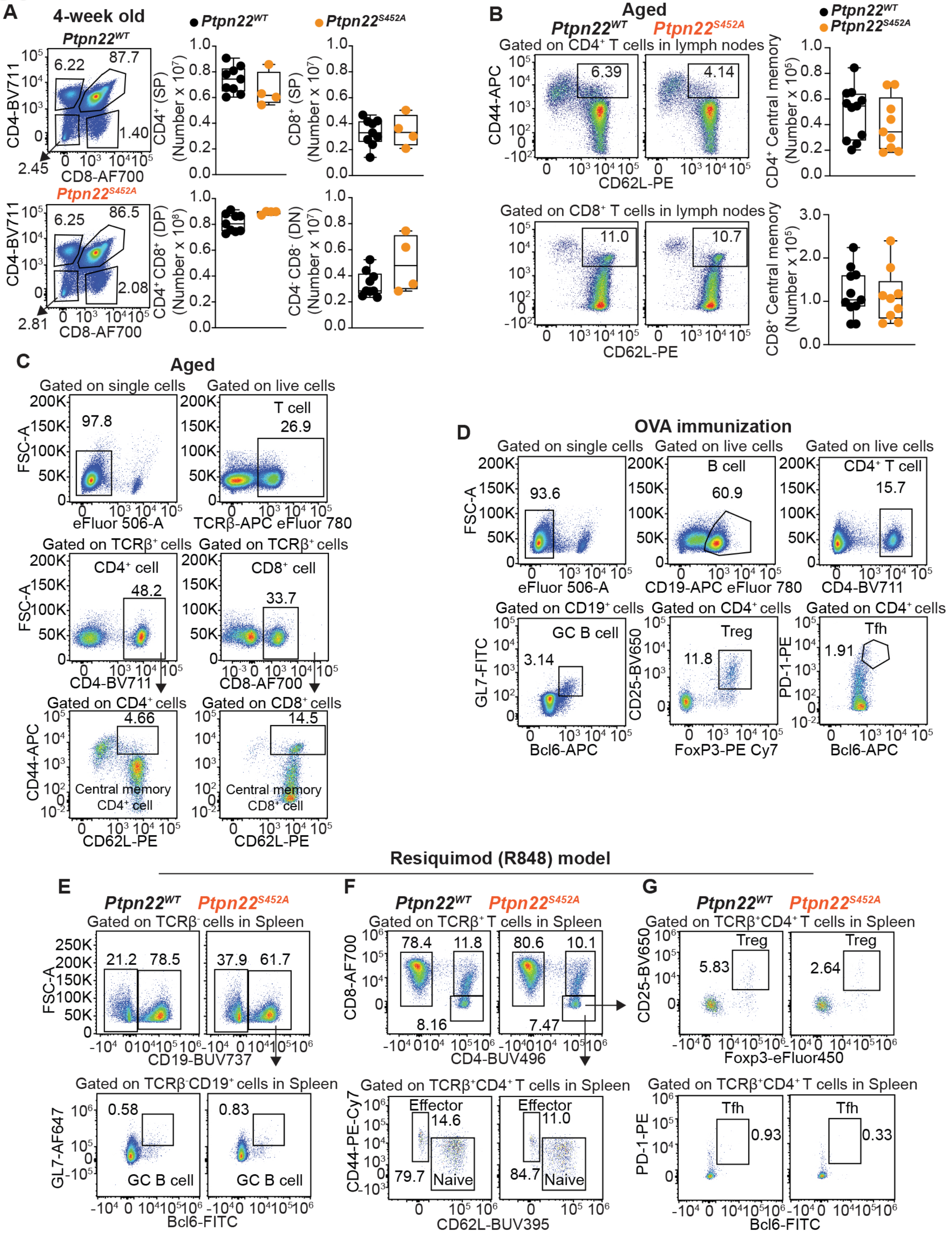

A
4-week old
Ptpn22WT
Ptpn22S452A
CD4-BV711
CD8-AF700
CD4+ (SP) (Number x 10^7)
CD8+ (SP) (Number x 10^7)
CD4+CD8+ (DP) (Number x 10^8)
CD4-CD8- (DN) (Number x 10^7)
B
Aged
Gated on CD4+ T cells in lymph nodes
Gated on CD8+ T cells in lymph nodes
CD44-APC
CD62L-PE
CD4+ Central memory (Number x 10^5)
CD8+ Central memory (Number x 10^5)
C
Aged
Gated on single cells
Gated on live cells
T cell
eFluor 506-A
TCRβ-APC eFluor 780
Gated on TCRβ+ cells
CD4+ cell
CD8+ cell
CD4-BV711
CD8-AF700
Gated on CD4+ cells
Gated on CD8+ cells
Central memory CD4+ cell
Central memory CD8+ cell
CD44-APC
CD62L-PE
D
OVA immunization
Gated on single cells
Gated on live cells
B cell
CD4+ T cell
eFluor 506-A
CD19-APC eFluor 780
CD4-BV711
Gated on CD19+ cells
Gated on CD4+ cells
GC B cell
Treg
Tfh
GL7-FITC
CD25-BV650
PD-1-PE
Bcl6-APC
FoxP3-PE Cy7
Resiquimod (R848) model
E
Gated on TCRβ- cells in Spleen
CD19-BUV737
Gated on TCRβ-CD19+ cells in Spleen
GL7-AF647
Bcl6-FITC
GC B cell
F
Gated on TCRβ+ T cells in Spleen
CD8-AF700
CD4-BUV496
Gated on TCRβ+CD4+ T cells in Spleen
CD44-PE-Cy7
CD62L-BUV395
Effector
Naive
G
Gated on TCRβ+CD4+ T cells in Spleen
CD25-BV650
Foxp3-eFluor450
Treg
Tfh
PD-1-PE
Bcl6-FITC
FSC-A

fig. S8. Immunophenotyping results for *Ptpn22^{WT}* vs *Ptpn22^{S452A}* mice.

(**A**) Thymocyte populations of 4-week-old *Ptpn22^{WT}* (n=9) or *Ptpn22^{S452A}* (n=4) mice. Single-positive (SP, $CD4^+$ or $CD8^+$), double-positive (DP, $CD4^+$ $CD8^+$), or double-negative (DN, $CD4^-$ $CD8^-$) populations were quantified as number of thymocytes. (**B**) Total numbers of $CD4^+$ and $CD8^+$ T cell with phenotype of central memory ($CD44^{hi}$ $CD62L^{hi}$) cells in lymph nodes of aged (over 6-month) *Ptpn22^{WT}* (n=11) or *Ptpn22^{S452A}* (n=9) mice. (**C**) Representative gating strategy to quantify central memory T cells in spleen (shown) or lymph nodes of aged (>6 months) *Ptpn22^{WT}* or *Ptpn22^{S452A}* mice. (**D**) Representative gating strategy to quantify GC B cell, Tfh, and Treg populations in draining lymph nodes of *Ptpn22^{WT}* or *Ptpn22^{S452A}* mice immunized with OVA protein. (**E**-**G**) Flow cytometry analysis of total B cell, GC B cell (**E**), T cell (**F**), Tfh and Treg cell (**G**) populations in spleens of *Ptpn22^{WT}* or *Ptpn22^{S452A}* mice treated with R848 at 28 days. Data are presented as mean ± SEM (**A** and **B**).

Table S1. SLE patients and healthy control PBMC

Types	**ID**	**Sex**	**SLEDAI**
SLE	P2123	Female	4
	P2110	Female	6
	P2115	Male	2
	P2124	Female	4
	P2122	Female	4
	P2114	Female	9
Healthy control	C0145	Female	/
	C0146	Female	/
	C0147	Female	/
	C0149	Female	/

Table S2. Primers used in the study

Name	Sequences
pEF3-S449A-F	CACGGACCAAAGCAACTCCTTTTGA
pEF3-S449A-R	TCAAAAGGAGTTGCTTTGGTCCGTG
pEF3-S449E-F	CACGGACCAAAGAGACTCCTTTTGA
pEF3-S449E-R	TCAAAAGGAGTCTCTTTGGTCCGTG
pEF3-S449D-F	CACGGACCAAAGACACTCCTTTTGA
pEF3-S449D-R	TCAAAAGGAGTGTCTTTGGTCCGTG
HDR 449-830bp F	TTTTAGTACCACAAAAGCAGCAAAA
HDR 449-830bp R	ACTTACCTAGTACAGCTGACTCCTGG
449A-563-F (Screening)	AGTACCACAAAAGCAGCAAAA
449A AS-R (Allele specific)	CAATTCAAAAGGAGTGGC
449A-593-F (CspCI digestion)	GGTGTCATCATTACGCTTTC
449A-593-R (CspCI digestion)	TGGTGCTTTACATTAGAGGC

Table S3. Human PTPN22 optimized CDS sequences

Name	Optimized CDS sequences
Optimized WT	ACCTCCGAGCCCAAGAAGTTCGACGATTCTGTGATCCTGCGGCCATCCAAGT<br>CTGTGAAGCTGAGATCCCCCAAGTCTGAGCTGCACCAGGACCGGAGCAGCC<br>CACCTCCACCACTGCCTGAGAGAACACTGGAGAGCTTCTTTCTGGCCGACGA<br>GGATTGCATGCAGGCCCAGTCCATCGAGACCTACAGCACATCCTATCCTGAT<br>ACAATGGAGAACAGCACATCTAGCAAGCAGACCCTGAAGACACCAGGCAA<br>GAGCTTTACCAGGTCTAAGAGCCTGAAGATCCTGCGCAATATGAAGAAGTC<br>TATCTGCAACAGCTGTCCTCCAAATAAGCCCGCCGAGAGCGTGCAGTCCAA<br>CAATTCCTCTAGCTTCCTGAACTTCGGCTTTGCCAATCGGTTTTCCAAGCCCA<br>AGGGCCCTAGAAACCCCCCTCCAACATGGAATATCTGA
Optimized S449A	ACATCTGAGCCCAAGAAGTTCGATGACTCCGTGATCCTGAGGCCATCTAAGT<br>CCGTGAAGCTGAGGAGCCCTAAGTCTGAGCTGCACCAGGACAGAAGCTCCC<br>CACCCCCTCCACTGCCTGAGAGAACCCTGGAGTCCTTTTTCCTGGCCGATGA<br>GGACTGCATGCAGGCCCAGAGCATCGAGACATACTCCACAAGCTACCCTGA<br>CACAATGGAGAACTCCACCAGCTCCAAGCAGACACTGAAGACCCCAGGCAA<br>GAGCTTCACAAGGAGCAAGTCCCTGAAGATCCTGCGGAACATGAAGAAGAG<br>CATCTGCAACAGCTGTCCACCCAACAAGCCTGCCGAGTCCGTGCAGAGCAA<br>TAACTCTAGCTCCTTCCTGAATTTCGGCTTCGCCAACAGATTCTCCAAGCCTA<br>AGGGCCCTAGAAACCCACCTCCCACATGGAACATCTGA

Table S4. Jurkat cells used in the study

Name	Description
Jurkat $CD4^+$ T cell	Original Jurkat E6.1 cell
J.N22 KO	Jurkat cell with PTPN22 KO
J.N22 WT	Jurkat with 3× FLAG tag KI PTPN22 WT
J.N22 449	Jurkat with 3× FLAG tag KI PTPN22 S449A
JCaM1.6	Jurkat cell without LCK
J45.01	Jurkat cell without CD45
J.Csk^{AS}/CD45	Jurkat cell with a Csk analog-sensitive allele and CD45 knock out
P116	Jurkat cell without ZAP70
J.Lck WT	JCaM 1.6 cell reconstituted with LCK WT
J.Lck Y192F	JCaM 1.6 cell reconstituted with LCK Y192F
J.OT1. $hCD8^+$	Original J.OT1.$hCD8^+$ cells
J.OT1 N22 WT	J.OT1.$CD8^+$ Jurkat cell with PTPN22 WT
J.OT1 N22 449	J.OT1.$CD8^+$ Jurkat cell with PTPN22 S449A
J.OT1 N22 KO	J.OT1.$CD8^+$ Jurkat cell with PTPN22 KO
3× FLAG N22 J.OT1	J.OT1.$CD8^+$ Jurkat cell with 3× FLAG tag KI PTPN22

Fig. 1K

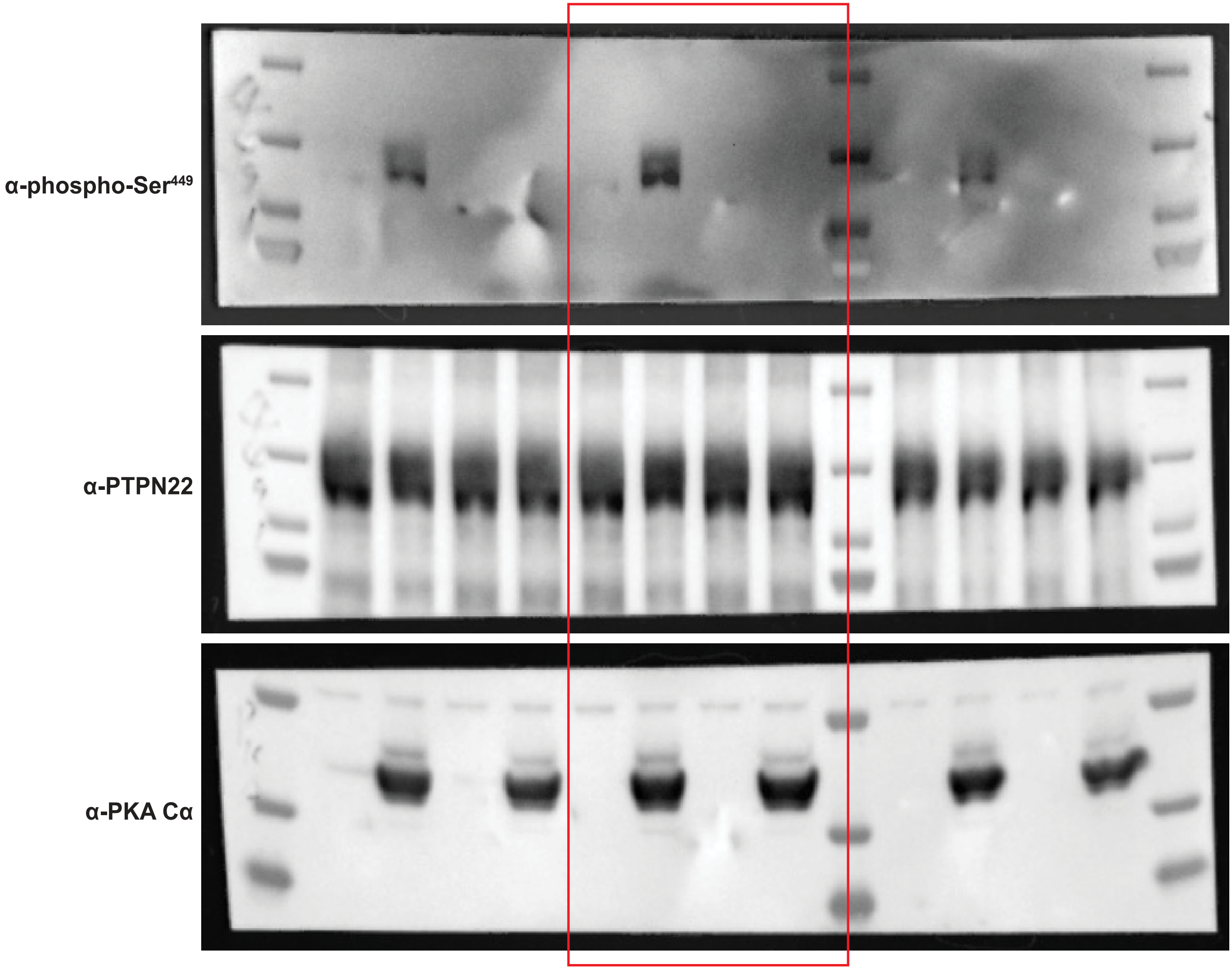

Fig. 3C

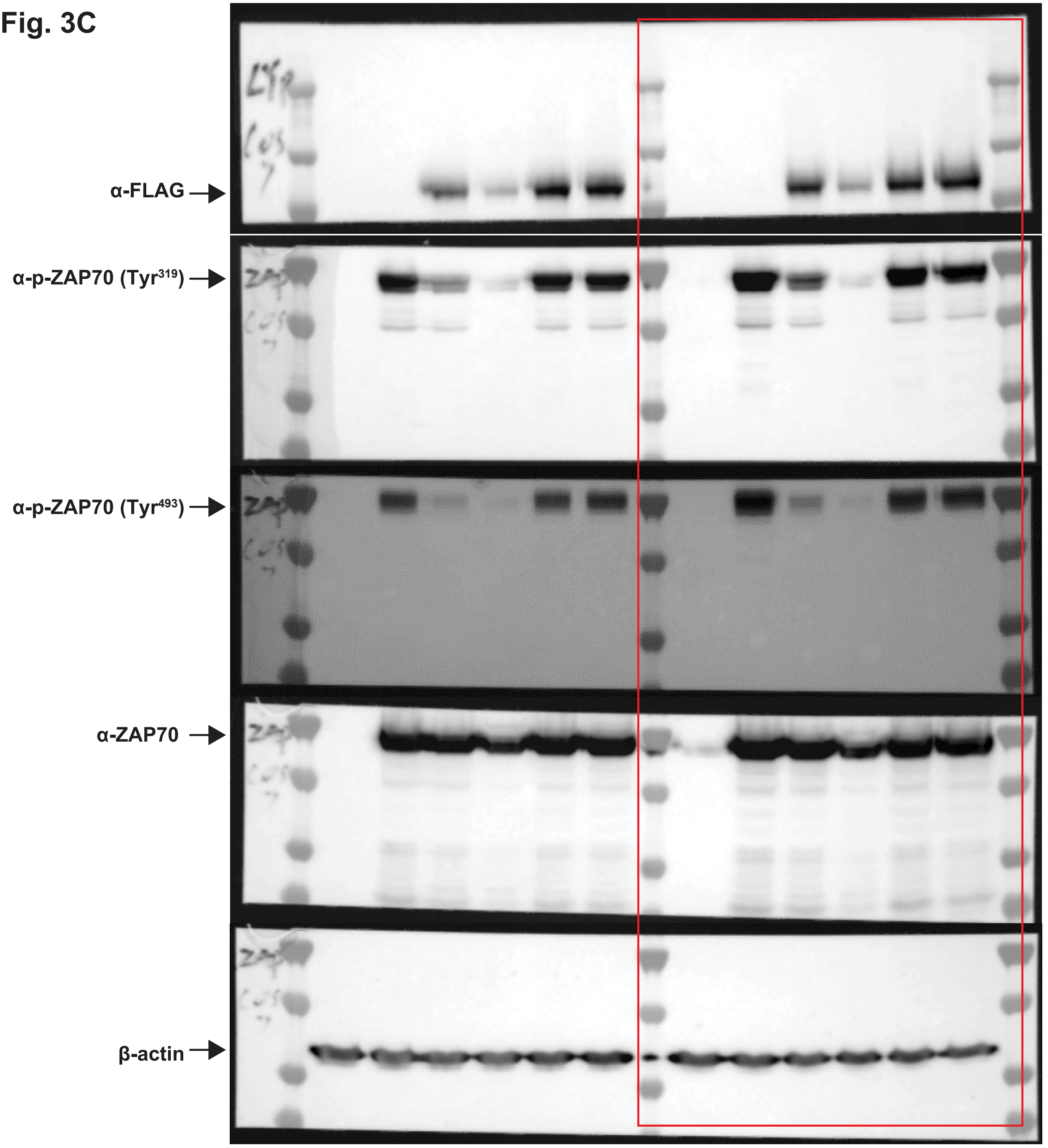

Fig. 4G

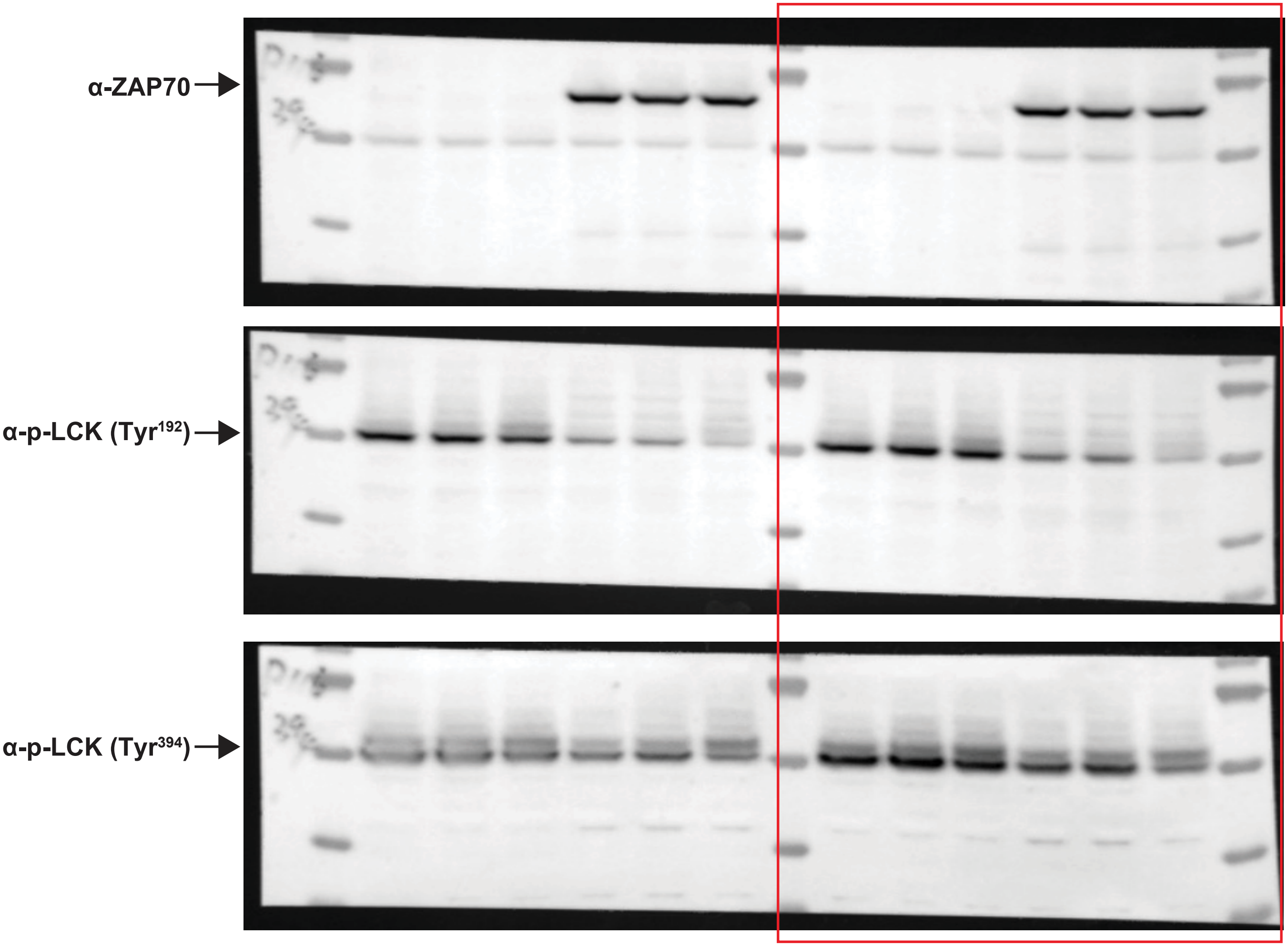

Fig. 4G

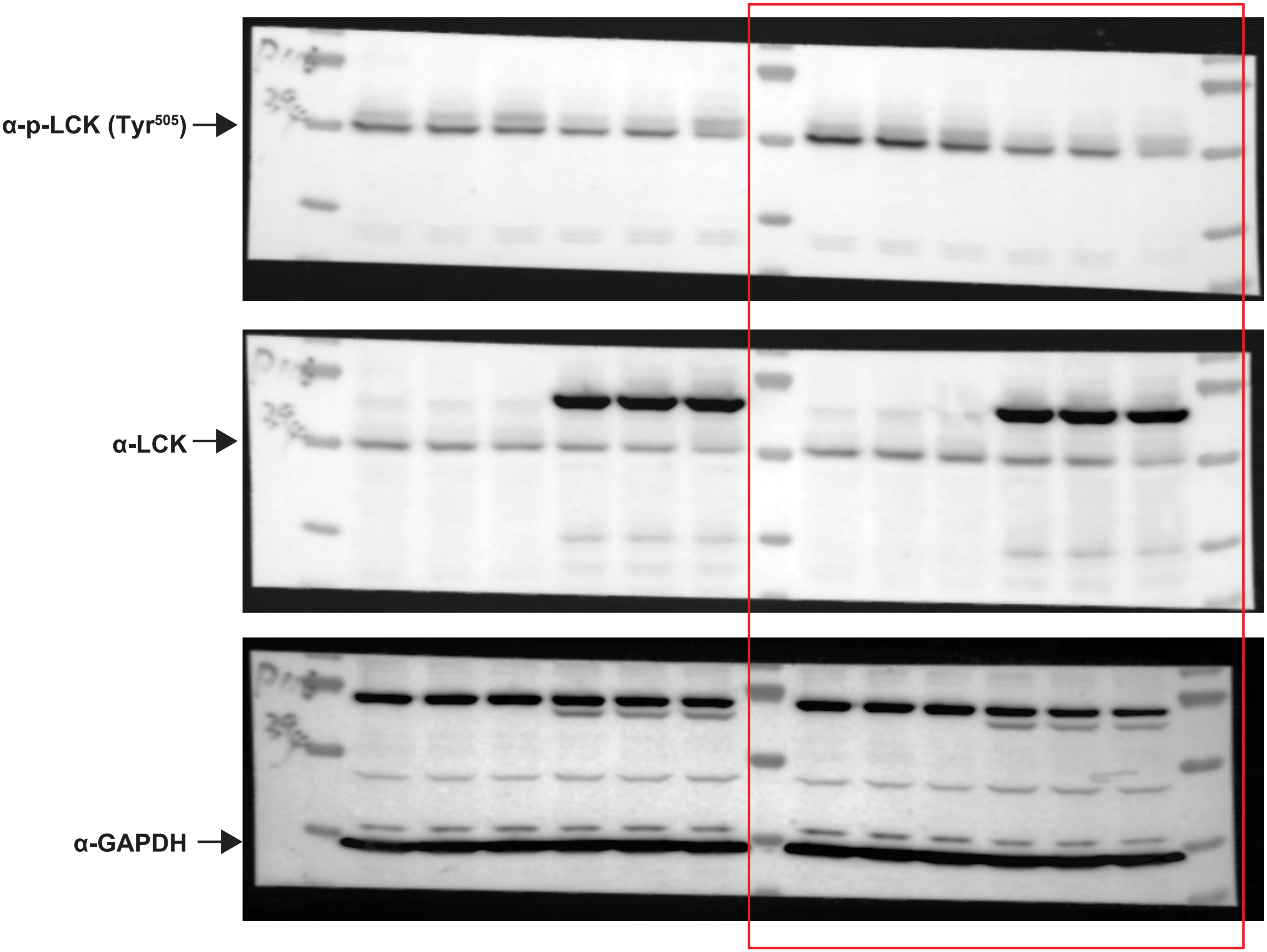

Fig. 5C

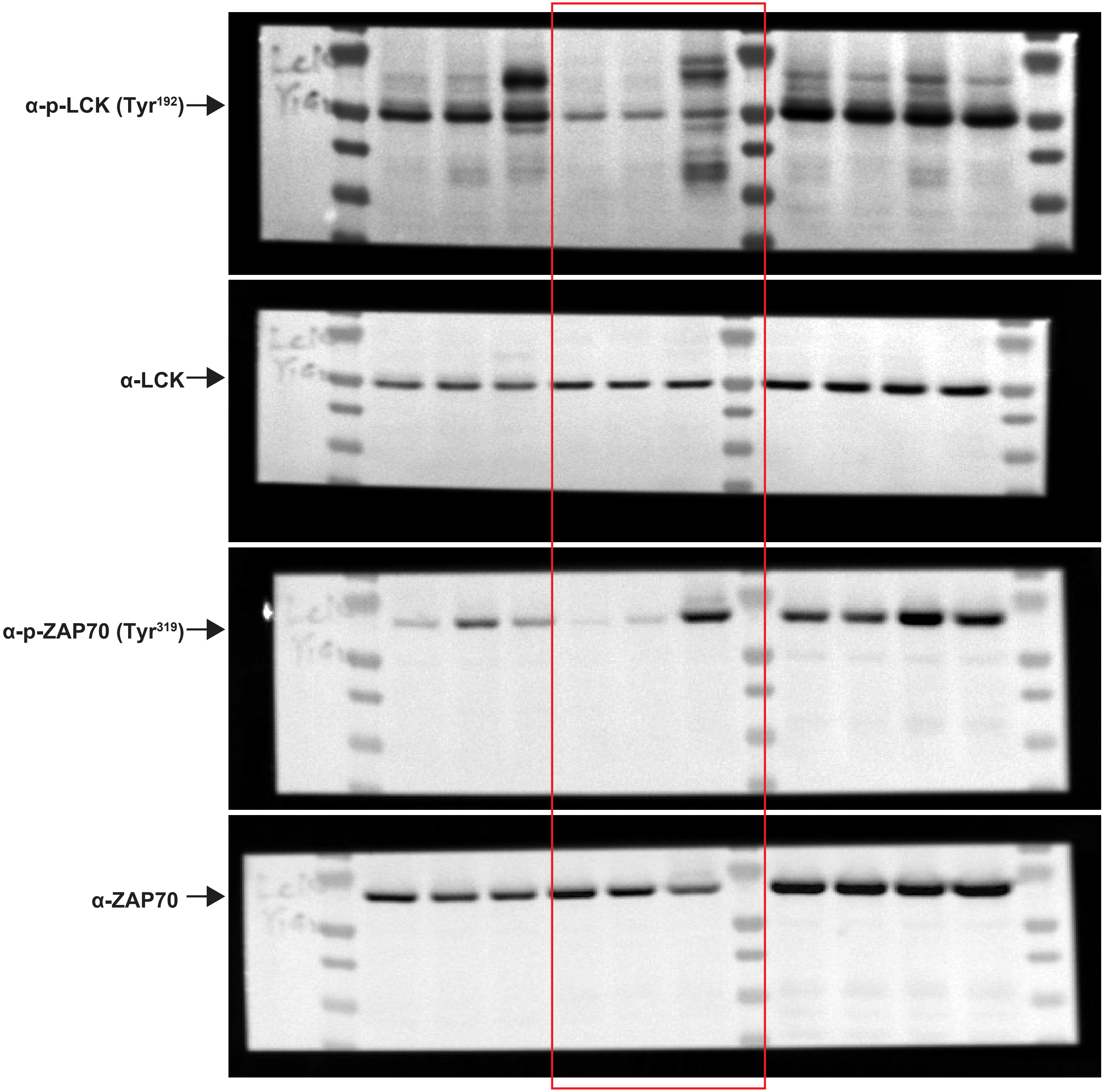

Fig. 5C

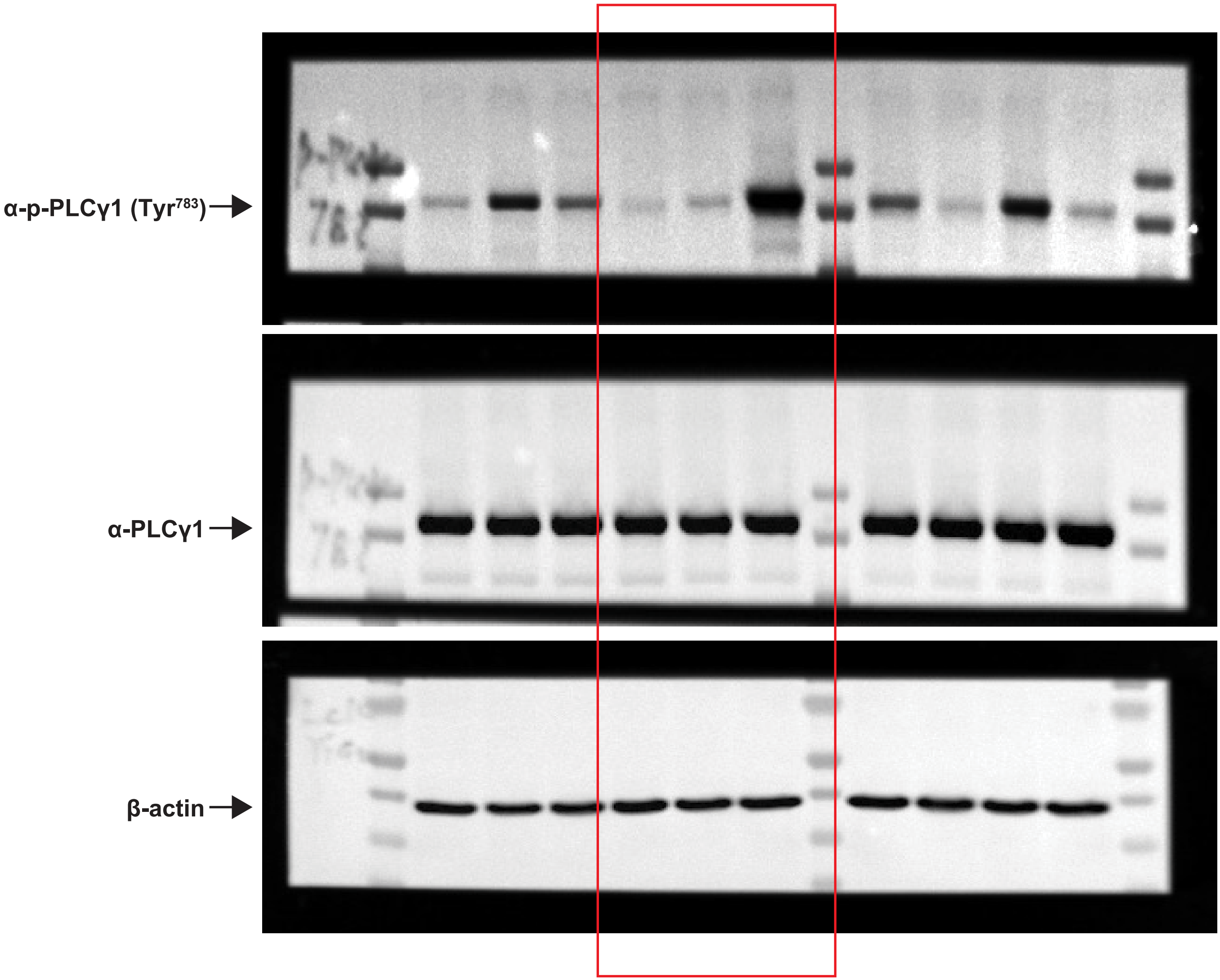

Supplement: Supplementary file 1 — Figs. S1 to S8 Tables S1 to S4 Uncropped blots in Figs. 1K, 3C, 4G, and 5C [file sciadv.adw2568_sm.pdf]
